# An improved temperature-sensitive shuttle vector system for scarless gene deletion in human-gut-associated *Bifidobacterium* species

**DOI:** 10.1016/j.isci.2024.111080

**Published:** 2024-10-01

**Authors:** Tomoya Kozakai, Aruto Nakajima, Keisuke Miyazawa, Yuki Sasaki, Toshitaka Odamaki, Toshihiko Katoh, Takeshi Fukuma, Jin-zhong Xiao, Tohru Suzuki, Takane Katayama, Mikiyasu Sakanaka

**Affiliations:** 1 Graduate School of Biostudies, Kyoto University, Kyoto 606-8502, Japan; 2 Faculty of Frontier Engineering, Kanazawa University, Kanazawa, Ishikawa 920-1192, Japan; 3 WPI Nano Life Science Institute (WPI-NanoLSI), Kanazawa University, Kanazawa, Ishikawa 920-1192, Japan; 4 Innovative Research Institute, R&D Division, Morinaga Milk Industry Co. Ltd., Zama, Kanagawa 252-8583, Japan; 5 Faculty of Applied Biological Sciences, Gifu University, Gifu 501-1193, Japan

**Keywords:** Molecular biology, Microbiology, Methodology in biological sciences

## Abstract

*Bifidobacterium* is a prevalent bacterial taxon in the human gut that comprises over 10 (sub)species. Previous studies suggest that these species use evolutionarily distinct strategies for symbiosis with their hosts. However, the underlying species-specific mechanisms remain unclear due to the lack of efficient gene knockout systems applicable across different species. Here, we developed improved temperature-sensitive shuttle vectors by introducing Ser139Trp into the replication protein RepB. We then used temperature-sensitive plasmids to construct a double-crossover-mediated scarless gene deletion system. The system was employed for targeted gene deletion in *Bifidobacterium longum* subsp. *longum*, *B. longum* subsp. *infantis*, *Bifidobacterium breve*, *Bifidobacterium adolescentis*, *Bifidobacterium kashiwanohense*, and *Bifidobacterium pseudocatenulatum*. Deletion of genes involved in capsular polysaccharide biosynthesis, aromatic lactic acid production, and sugar utilization resulted in the expected phenotypic changes in the respective (sub)species. The temperature-sensitive plasmids developed in this study will aid in deciphering the evolutionary traits of the human-gut-associated *Bifidobacterium* species.

## Highlights

• A temperature-sensitive (Ts) shuttle vector system for bifidobacteria was developed
• The Ts plasmid system was employed to develop a scarless gene knockout system
• Different genes in six *Bifidobacterium* (sub)species were successfully disrupted
• This method helps to clarify the bifidobacteria-host symbiosis at the molecular level

## Graphical abstract

**Figure:**
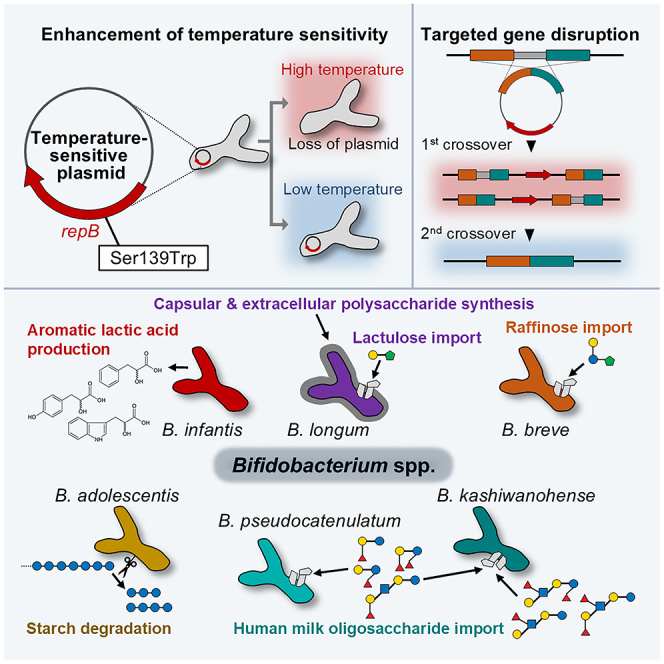

## Introduction

The genus *Bifidobacterium*, a prevalent bacterial taxon in the human gut, is positively associated with host health.^1^^,^^2^^,^^3^ To date, approximately 12 (sub)species of *Bifidobacterium* have been isolated from human feces.^4^^,^^5^^,^^6^^,^^7^ However, cross-sectional and longitudinal studies have revealed that the prevalence and abundance of each species vary not only inter-individually but also within an individual across different life stages, suggesting the presence of various strategies in this genus to adapt to the ever-changing gut environment.^4^^,^^8^^,^^9^ The ability of bifidobacteria to utilize nutrients such as sugars and to produce metabolites affecting the host health varies across all (sub)species, especially between infant- and adult-gut-associated *Bifidobacterium* species. Infant-gut-associated bifidobacteria, represented by *Bifidobacterium longum* subsp. *longum* (*B*. *longum*), *B. longum* subsp. *infantis* (*B*. *infantis*), *Bifidobacterium breve*, *Bifidobacterium pseudocatenulatum*, and *Bifidobacterium catenulatum* subsp. *kashiwanohense* (*B*. *kashiwanohense*), are characterized by the presence of specific pathways to utilize indigestible oligosaccharides contained in breastmilk (human milk oligosaccharides [HMOs]).^5^ Adult-gut-associated species, such as *Bifidobacterium adolescentis* and *B. catenulatum* subsp. *catenulatum*, can consume dietary oligo- and polysaccharides, but not HMOs.^5^^,^^10^^,^^11^^,^^12^^,^^13^^,^^14^ The ability to produce an immunomodulatory compound, indole lactic acid, has only been reported for infant-gut-associated bifidobacterial species.^8^^,^^15^ In contrast, the ability to produce gamma-aminobutyric acid, which modulates the gut-brain axis, is only observed in adult-gut-associated bifidobacterial species, such as *B. adolescentis*.^16^^,^^17^^,^^18^^,^^19^

Despite this, studies elucidating the molecular mechanisms underlying symbiotic interactions between bifidobacteria and hosts are limited to certain strains of *Bifidobacterium* species, including *B. breve*, *B. longum*, and *B. infantis*,^20^^,^^21^^,^^22^ owing to the lack of efficient gene disruption systems. Previous pioneering studies have established several gene disruption systems; however, these systems work only in specific strains (e.g., *B. breve* UCC2003, *B. longum* JCM 31944 [105-A], and *B. infantis* ATCC 15697^T^ [JCM 1222^T^]).^20^^,^^21^^,^^22^^,^^23^^,^^24^^,^^25^^,^^26^^,^^27^^,^^28^^,^^29^^,^^30^^,^^31^^,^^32^

**Figure 1:**
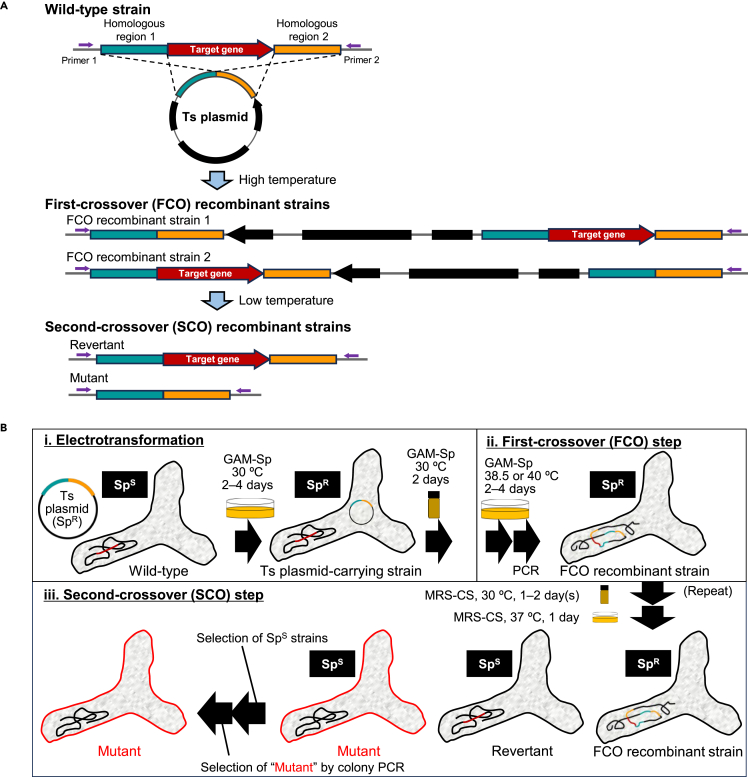
Experimental procedure for two-step double-crossover gene deletion using a Ts plasmid (A) A schematic representation of two-step double-crossover gene deletion using a Ts plasmid. As described in the introduction section, the Ts plasmid carrying the up- and downstream homologous regions of the target gene is replicable in the cells of (bifido)bacteria at low permissive temperatures, whereas increasing the temperature results in a cessation of plasmid replication and thus facilitates selection of recombinant strains carrying the Ts plasmid integrated at the chromosomal target locus via a first crossover (FCO) recombination event. Returning to permissive low temperatures re-initiates plasmid replication, which can lead to either targeted gene deletion or reversion to the wild-type (WT) genotype via a second crossover (SCO) recombination event. Note that the genotypes of WT, FCO, and SCO recombinant strains can be verified by PCR of the target gene locus using the primer pair indicated by purple arrows. (B) Experimental procedure for gene deletion. Ts plasmid carrying the up- and downstream regions of the target gene was introduced into bifidobacterial cells by electroporation (i). The resulting Sp^R^ transformant cells were incubated at 38.5°C or 40°C on GAM-Sp plate to obtain FCO recombinant strains (ii). FCO recombinant strains were then subcultured at 30°C in antibiotic-free liquid MRS-CS medium to induce SCO recombination (iii). The resulting propagated cells possibly contain the plasmid-cured SCO recombinant strains, and therefore, subsequent cultivation was performed at 37°C (optimal temperature for bifidobacteria). The mutants were then screened by phenotypic examination of Sp sensitivity, followed by colony PCR of the target gene locus.

Temperature-sensitive (Ts) plasmid has been widely used for two-step double-crossover-mediated gene deletion in different bacterial species/strains, including genetically intractable species and strains.^33^^,^^34^^,^^35^^,^^36^ The Ts plasmid, which typically carries the upstream and downstream homologous regions of the target gene, is generally replicable in the cells of certain bacteria at low permissive temperatures, such as 30°C, whereas increasing the temperature results in a cessation of plasmid replication and thus efficient selection of recombinant strains carrying the Ts plasmid integrated at the chromosomal target locus via a first crossover (FCO) recombination event (Figure 1A). Returning to permissive temperatures re-initiates plasmid replication, which can lead to either targeted gene deletion or reversion to the wild-type (WT) genotype via a second crossover (SCO) recombination event (see Figure 1A).

Here, we developed an efficient Ts-plasmid-based scarless gene deletion system that works across different *Bifidobacterium* (sub)species. We aimed to improve the Ts phenotype exerted by the previously constructed *Escherichia coli-Bifidobacterium* shuttle vector,^28^^,^^37^ as the Ts phenotype is observed at the temperature closest to the upper limit of cell growth (∼42°C). A plasmid with a strong Ts phenotype at ∼40°C was then used to construct scarless deletion mutants of six (sub)species of human-gut-associated *Bifidobacterium*, as indicated by the workflow shown in Figures 1A and 1B. The phenotypic changes in the resulting mutants were examined to ascertain the physiological functions of the disrupted target genes. Our scarless gene deletion system can aid in elucidating the molecular mechanisms by which different *Bifidobacterium* species adapt to different niches.

## Results

### Replication initiation protein RepB with S139W substitution exhibits enhanced Ts phenotype in *B. longum*

Plasmid pKO403, which carries a mutated *repB* with S139I substitution, exhibits a Ts phenotype when *B. longum* JCM 31944 is used as the host strain and grown on the de Man, Rogosa, and Sharpe (MRS) medium plates.^28^ However, this phenotype appears only at ≥ 42°C, which is almost the highest limit temperature for cell proliferation. Therefore, in this study, we attempted to enhance the Ts phenotype conferred by the plasmid.

**Figure 2:**
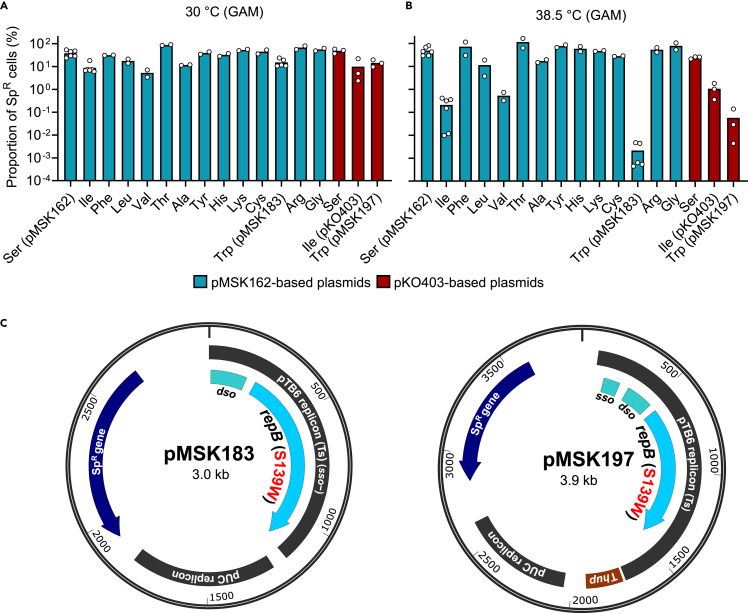
Replication initiation protein RepB with S139W substitution leads to an enhanced Ts phenotype in *B. longum* JCM 31944 (A and B) Effect of the 139^th^ amino acid residue of RepB on plasmid stability at 30°C (A) or 38.5°C (B). Ser-139 of the *E. coli-Bifidobacterium* shuttle vector, pMSK162 (blue bar; Figure S1), was replaced with the remaining 19 amino acid residues by site-directed mutagenesis. The resulting plasmids and parental plasmid pMSK162 were introduced into *B. longum*, and the obtained 14 transformant cells were then spread onto GAM and GAM-Sp plates. The plates were incubated at 30°C or 38.5°C to determine the CFU. Plasmid stability is shown as the percentage of Sp^R^ cells in the total number of cells. Similar experiments were performed for pKO403 and its derivatives (red bar; Figure S1). Data are represented as dot plots with mean of at least biological duplicates. (C) Schematic maps of the *E*. *coli*-*Bifidobacterium* shuttle vectors, pMSK183 and pMSK197. pMSK183 and pMSK197, which are derivatives of pMSK162 and pKO403 (Figure S1), respectively, harbor mutated *repB* (S139W). pTB6 replicon, replicon for bifidobacteria; *sso*, single-strand origin for replication; *dso*, double-strand origin for replication; *repB*, replication initiation protein gene; T*hup*, transcription terminator of *hup* gene from *B. longum*; pUC replicon, replicon for *E. coli*; Sp^R^ gene, spectinomycin resistance gene. Open-reading frames are indicated by arrows. Plasmid maps were drawn using SnapGene Viewer 7.0.1. See also Figures S1 and S2.

Assuming that the amino acid residue at position 139 of RepB governs the Ts phenotype of the replication, Ser-139 of an *E. coli*-*Bifidobacterium* shuttle vector, pMSK162 (see STAR Methods section; Figure S1), was replaced with the remaining 19 amino acid residues by site-directed mutagenesis. The resulting plasmids were introduced into *B. longum* JCM 31944 to examine their stability. Among the 19 plasmid variants, 13 variants with Arg-, Lys-, His-, Thr-, Cys-, Gly-, Ala-, Val-, Leu-, Ile-, Phe-, Tyr-, and Trp-substitutions conferred the spectinomycin (Sp)-resistant (Sp^R^) phenotype to *B. longum* grown on the Gifu anaerobic medium (GAM) plate incubated at 30°C. No transformants were obtained from the other six plasmid variants. Transformants obtained for the 13 plasmid variants were suspended in 0.85% (w/v) NaCl, and the suspensions were spread onto both GAM and GAM-Sp plates after serial dilution. The plates were incubated at 30°C or 38.5°C to determine the colony-forming unit (CFU) (Figures 2A and 2B). When incubated at 30°C, CFU of *B. longum* on GAM-Sp plates decreased by 1.1- to 19-fold compared to that when incubated on the antibiotic-free GAM plate (total *B. longum*), indicating the intrinsic instability of the plasmids.^38^ A similar decrease in CFU on the GAM-Sp plate was observed for the strain carrying the parental plasmid, pMSK162. Interestingly, drastic CFU change was observed when the plates were incubated at 38.5°C. CFU on GAM-Sp plates of *B. longum* carrying the plasmid with S139W substitution dropped by 4.7 × 10^4^-fold compared to that on antibiotic-free GAM plates. A reduction in CFU was also observed for *B. longum* strains carrying the plasmid with the S139I or S139V substitution, albeit to a lesser extent. The other plasmids were maintained at a similar level as that observed when incubated at 30°C. S139W substitution also decreased the stability of pKO403 (Figure S1)^28^ by 1.7 × 10^3^-fold at non-permissive temperature (Figures 2A and 2B), but no obvious effect was observed compared to the parental plasmid at the permissive temperature. pMSK162 and pKO403 with the S139W replacement are hereafter referred to as pMSK183 and pMSK197, respectively (Figure 2C). Notably, Ts phenotype of these strains at 38.5°C was observed only when GAM plates were used for CFU determination and never observed with MRS plates supplemented with 0.02% (w/v) cysteine-HCl and 0.34% (w/v) sodium ascorbate (CS) (Figure S2). However, the reason for this medium-dependent phenotypic discrepancy remains unclear. GAM was supplemented with 0.3% glucose, whereas MRS-CS was supplemented with 2% glucose, which affected the growth of bifidobacteria and possibly RepB expression. These results suggest that, although both GAM and MRS-CS show relatively stable replication of the Ts plasmids under permissive temperatures (e.g., 30°C), GAM is better suited than MRS-CS for examining the Ts phenotype conferred by the plasmid under non-permissive temperatures (e.g., 38.5°C).

### Scarless gene deletion in *B. longum* using Ts plasmids

Two Ts plasmids, pMSK183 and pMSK197, were used for a two-step double-crossover-mediated gene deletion (Figure 1A) in *B. longum* JCM 31944, to compare their efficiencies. The parental plasmid, pKO403, was included in the analysis. We chose *cpsD* (BL105A_0405) as the disruption target, which encodes a priming glycosyltransferase (Figure S3) because the mutant could be visually identified on agar plates as smaller and thinner colonies than the WT colonies due to reduced production of capsular and extracellular polysaccharides (CPSs/EPSs).^39^ Plasmid construction, subsequent cultivation, and confirmation procedures are shown in Figure 1B (see the STAR Methods section). As mentioned earlier, Sp-supplemented GAM was used to select the FCO recombinant strains (Figure 1B(i and ii)) at the non-permissive temperature (38.5°C or 40°C). The selected strains were then cultivated in MRS-CS liquid medium, in which bifidobacteria show faster growth than in GAM,^18^ at the permissive temperature to allow for SCO recombination to occur (Figure 1B(iii)). Subsequently, a portion of the culture was spread onto MRS-CS plates, and the plates were incubated at 37°C (Figure 1B(iii)).

**Figure 3:**
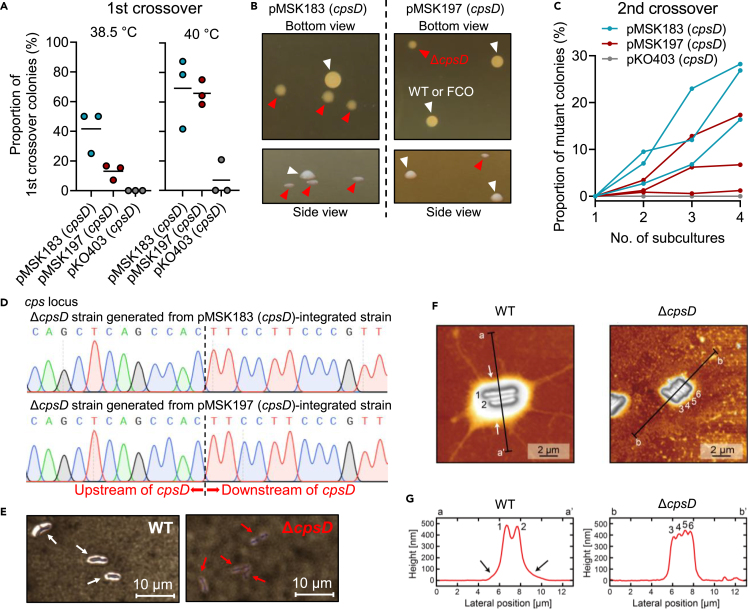
Verification of Ts-plasmid-based scarless gene deletion in *B. longum* JCM 31944 (A) Number of first crossover (FCO) recombinant strains in the total population when the transformants carrying pMSK183 (*cpsD*), pMSK197 (*cpsD*), or pKO403 (*cpsD*) were incubated at non-permissive temperatures (38.5°C or 40°C) on the GAM-Sp plate. FCO recombination was verified by colony PCR as shown in Figure S4A. Data are represented as dot plots with mean of biological triplicates. (B) Colony morphology of Δ*cpsD* grown on agar plates is smaller and thinner than that of the wild-type (WT) and FCO recombinant strains, making the Δ*cpsD* strain visually distinguishable from the other strains. Images were obtained from bottom and side views of the agar plates. (C) Number of second crossover (SCO) recombinant Δ*cpsD* mutants in the total population when the FCO recombinant strains were incubated on the antibiotic-free MRS-CS plate. Prior to bacterial inoculation on the plate, FCO recombinant strains were subcultured 1–4 times at 30°C in the antibiotic-free MRS-CS liquid medium to induce SCO recombination. Data are shown for each replicate of biological triplicates. (D) Waveform data obtained by Sanger sequencing of the Δ*cpsD* locus. The Δ*cpsD* locus was PCR-amplified using the genome of mutants generated from pMSK183 (*cpsD*)- or pMSK197 (*cpsD*)-integrated FCO strains. Dotted lines indicate the boundary between upstream and downstream regions of *cpsD*. (E) Optical microscopy images of the bifidobacterial cells. WT (left panel) and Δ*cpsD* (right panel) cells were subjected to India ink staining followed by Gram staining. A clear halo corresponding to CPS/EPS was observed around the cells of WT, but not Δ*cpsD*. (F and G) Atomic force microscopy (AFM) images of bifidobacterial cells on a glass substrate in air. Height images of WT (left panel) and Δ*cpsD* (right panel) cells are shown in (F). Line profiles taken along line a-a’ or b-b’ in (F) are shown in (G). Numbers and arrows on the line profiles correspond to those in (F). See also Figures S3, S4, and S9.

When cultivated at 38.5°C (Figure 1B(ii)), FCO recombination at the *cpsD* locus occurred at 42% and 13% of Sp^R^ colonies obtained for the pMSK183 (*cpsD*)- and pMSK197 (*cpsD*)-introduced strains, respectively, whereas no intended FCO recombination was detected among the pKO403 (*cpsD*)-introduced Sp^R^ colonies (Figures 3A and S4A). The efficiency of FCO recombination increased to ∼70% of Sp^R^ colonies obtained for pMSK183 (*cpsD*)- and pMSK197 (*cpsD*)-introduced strains when the cultivation was done at 40°C (Figures 3A and S4A). The plasmid integration at the *cpsD* locus also occurred when pKO403 (*cpsD*)-introduced strains were incubated at 40°C though the efficiency was less than 7% of the Sp^R^ population.

Strains with the plasmid integration at the *cpsD* locus were then subjected to SCO recombination to generate Δ*cpsD* mutants (Figure 1B(iii)). The Δ*cpsD* colonies were visually distinguishable from FCO and WT (revertant) colonies as mentioned earlier (Figure 3B), some of which were further analyzed by a colony PCR to examine genotype-phenotype accordance (Figure S4B). The number of the Δ*cpsD* mutants in the total populations increased when the subculture was repeated up to four times. Specifically, the percentile of the Δ*cpsD* strains among the total populations increased to 16%–28% and 1%–17% after four passages of cultures for the pMSK183 (*cpsD*)-integrated and the pMSK197 (*cpsD*)-integrated strains, respectively, in the three independent experiments (Figure 3C). By contrast, no Δ*cpsD* strain was obtained from the strains carrying the chromosomal integration of pKO403 (*cpsD*) in our experiments (Figure 3C). In either case in which pMSK183 (*cpsD*) or pMSK197 (*cpsD*) was used for a Ts plasmid, the scarless excision of Ts plasmids from the target locus occurred, as revealed by Sanger sequencing of the amplified fragments at the region (Figure 3D). India ink staining followed by Gram staining visualized clear haloes (i.e., a CPS/EPS-like extracellular layer) around the cells of WT (Figure 3E), whereas no such clear haloes were observed for Δ*cpsD* cells.^39^ Atomic force microscopy (AFM) analysis further corroborated the reduced CPS/EPS-like extracellular layer of the Δ*cpsD* cells (Figures 3F and 3G). The lateral length of the extracellular layer was ∼1–2 μm for WT cells but was ∼0.5 μm for Δ*cpsD* cells (Figure 3F). The cell height of the Δ*cpsD* strain decreased more sharply near the edge of the cells compared with that of the WT strain (Figure 3G). These results indicate that the Ts plasmids, pMKS183 and pMSK197, serve as efficient tools for scarless gene deletion in *B. longum*.

### Scarless gene deletion in other *Bifidobacterium* species using pMSK197

We further examined whether our Ts plasmid system is effective in other *Bifidobacterium* species. pMSK197 was used for the experiments because we found that *B. longum* carrying pMSK197 grew faster than *B. longum* carrying pMSK183, with generation times differing by 1.5-fold (2.9 h versus 4.3 h) at 30°C (Figures S5A and S5B). Genes with different metabolic functions, ranging from sugar utilization to aromatic lactic acid production, and with different lengths, ranging from 1.0 kb to 5.3 kb, were chosen for the targets (Table 1; Figure S3). Specifically, Blon_1090 (*aldh*) and Blon_1092, which encode an aromatic lactate dehydrogenase^8^ and a putative amino acid aminotransferase, respectively, in *B. infantis* JCM 1222^T^, BAD_0708 (*pulP*) encoding an extracellular type II pullulanase in *B. adolescentis* JCM 1275^T^,^40^ BBKW_1838 encoding a solute-binding protein (SBP) of fucosyllactose transporter-2 cluster III (FL2-III) in *B. kashiwanohense* JCM 15439^T^,^41^ BBPC_1775–1777 encoding a fucosyllactose transporter-2 cluster I (FL2-I) in *B. pseudocatenulatum* JCM 1200^T^,^41^ and BBBR_1864–1866 (*rafBCD*) encoding a raffinose transporter in *B. breve* JCM 1192^T^^42^ were selected. *B. longum* JCM 31944 was also included, but a different gene, BL105A_0502, which encodes an SBP of the lactulose transporter,^43^ was chosen. To the best of our knowledge, to date, no targeted gene disruption has been reported for *B. adolescentis*, *B. kashiwanohense*, and *B. pseudocatenulatum*.

**Table 1:** Efficiency of FCO and SCO recombination in different *Bifidobacterium* species

Strain	Target gene	Gene function	Vector type	Efficiency of FCO recombination^a^	Efficiency of SCO recombination^a^			
					Proportion of Sp^s^ cells	Proportion of mutants	Proportion of revertants	No. of subcultures
*B. longum* subsp. *infantis* JCM 1222^T^	Blon_1090 (*aldh*)	Aromatic lactate dehydrogenase	pMSK197	60.0% (3/5)	2.9% (3/102)	66.7% (2/3)	33.3% (1/3)	3
	Blon_1092	Putative amino acid aminotransferase	pMSK197	100% (2/2)	5.1% (16/312)	68.8% (11/16)	31.2% (5/16)	4
*B. adolescentis* JCM 1275^T^	BAD_0708 (*pulP*)	Extracellular type II pullulanase	pMSK197	86.7% (13/15)	5.0% (7/140)	57.1% (4/7)	42.9% (3/7)	1
*B. catenulatum* subsp. *kashiwanohense* JCM 15439^T^	BBKW_1838 (FL2-III)	SBP of FL transporter-2 cluster III	pMSK197	100% (2/2)	5.7% (12/210)	8.3% (1/12)	91.7% (11/12)	2
*B. pseudocatenulatum* JCM 1200^T^	BBPC_1775–1777 (FL2-I)	FL transporter-2 cluster I	pMSK197	75.0% (18/24)	77.8% (281/361)	31.3% (5/16)	68.8% (11/16)	2
*B. breve* JCM 1192^T^	BBBR_1864–1866 (*rafBCD*)	Raffinose transporter	pMSK197	18.8% (3/16)	5.3% (4/75)	50.0% (2/4)	50.0% (2/4)	2
*B. longum* subsp. *longum* JCM 31944^b^	BL105A_0502	SBP of lactulose transporter	pMSK197	100% (1/1)	1.8% (3/167)	33.3% (1/3)	66.7% (2/3)	3

a FCO: first crossover; SCO: second crossover.

b See also Figures 3A and 3C for BL105A_0405 (*cpsD*) in *B. longum* subsp. *longum* JCM 31944.

Upstream and downstream homologous regions of the target genes were amplified and individually inserted into pMSK197, and the resulting plasmids were introduced into the corresponding *Bifidobacterium* species. After incubation at 30°C, Sp^R^ transformants were obtained with transformation efficiencies of > 10^3^ CFU/μg DNA for *B. infants*, *B. kashiwanohense*, *B. breve*, and *B. longum* and of <10 CFU/μg DNA for *B. adolescentis* and *B. pseudocatenulatum*. Cultivation of the transformants at the non-permissive temperature on the GAM-Sp plate caused the intended FCO recombination in 3 out of 5 selected Sp^R^ *B. infantis* transformants for *aldh*, 2 out of 2 for Blon_1092, 13 out of 15 for *B. adolescentis*, 2 out of 2 for *B. kashiwanohense*, 18 out of 24 for *B. pseudocatenulatum*, 3 out of 16 for *B. breve*, and 1 out of 1 for *B. longum*, which was confirmed by colony or genomic PCR (Table 1; Figure S6). After incubation of the plasmid-integrated strains at the permissive temperature, Sp^S^ colonies that possibly include WT (revertant) and deletion mutants were obtained with varied efficiencies: 2.9% (3/102 colonies for *aldh*) and 5.1% (16/312 for Blon_1092) for *B. infantis*, 5.0% for *B. adolescentis* (7/140), 5.7% for *B. kashiwanohense* (12/210), 77.8% for *B. pseudocatenulatum* (281/361), 5.3% for *B. breve* (4/75), and 1.8% for *B. longum* (3/167) (Table 1). Following colony PCR analysis, deletion mutants were obtained from the pool of Sp^S^ colonies: 2 out of 3 (for *aldh*) and 11 out of 16 (for Blon_1092) for *B. infantis*, 4 out of 7 for *B. adolescentis*, 1 out of 12 for *B. kashiwanohense*, 5 out of 16 for *B. pseudocatenulatum*, 2 out of 4 for *B. breve*, and 1 out of 3 for *B. longum*. Scarless excision at the loci was confirmed by Sanger sequencing of the PCR products of the target regions (Figures S6 and S7). These results demonstrate that the Ts plasmid, pMSK197, is applicable to at least six (sub)species among the 12 human-gut-associated *Bifidobacterium* (sub)species.

### Phenotypic characterization of the *Bifidobacterium* deletion mutants

#### Aromatic lactate dehydrogenase and putative amino acid aminotransferase of *B. infantis* JCM 1222^T^

**Figure 4:**
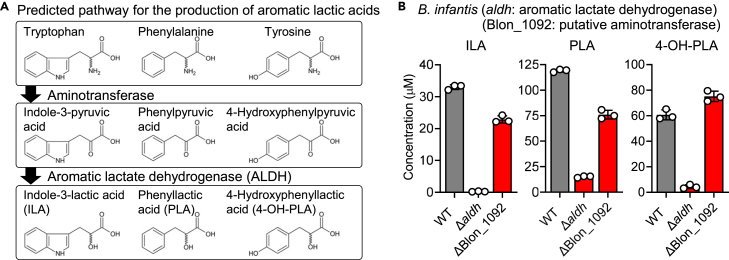
Production of aromatic lactic acids in *B. infantis* is primarily dependent on the aromatic lactate dehydrogenase gene *aldh* (A) Predicted pathway for the production of aromatic lactic acids (indole-3-lactic acid [ILA], phenyllactic acid [PLA], and 4-hydroxyphenyllactic acid [4-OH-PLA]). (B) Concentrations of aromatic lactic acids in the culture supernatants of WT, Δ*aldh*, and ΔBlon_1092 *B infantis*. Supernatants were collected after 24 h of incubation at 37°C in MRS-CS medium containing 2% glucose and subjected to LC-MS/MS analysis. Data are represented as dot plots with the mean ± standard deviation (SD) of biological triplicates. See also Figures S3 and S5–S7.

Indole-3-lactic acid (ILA), phenyllactic acid (PLA), and 4-hydroxyphenyllactic acid (4-OH-PLA) are gut microbial metabolites relevant to host health.^1^^,^^44^^,^^45^ In particular, ILA and PLA play important roles in maintaining the epithelial barrier and energy homeostasis in the host by acting as agonists of aryl hydrocarbon receptors and/or hydroxycarboxylic acid receptor 3.^45^^,^^46^ The anti-inflammatory effects of ILA have also been reported.^15^ We recently identified the gene encoding aromatic lactate dehydrogenase in *B. longum* and showed that the enzyme, which is distantly related to a canonical lactate dehydrogenase, is conserved only in infant-gut-associated *Bifidobacterium* species, among other gut microbes.^8^ PLA, 4-OH-PLA, and ILA can be synthesized from phenylalanine, tyrosine, and tryptophan, respectively, via a two-step reaction involving an amino acid aminotransferase(s) and aromatic lactate dehydrogenase (Figure 4A). The Δ*aldh* mutant of *B. infantis* JCM 1222^T^ completely lost an ILA-producing ability, whereas it produced PLA and 4-OH-PLA in small amounts (Figure 4B). We have no clear answer as to what enzymes other than aromatic lactate dehydrogenase catalyze the reaction in this species, but it has been reported that the canonical lactate dehydrogenase of lactobacilli can reduce phenylpyruvic acid through a side reaction.^47^ In contrast, deletion of the putative amino acid aminotransferase (Blon_1092), which comprises a gene cluster with *aldh*, did not largely affect the production of aromatic lactic acids (Figure 4B). The mutant synthesized 1.4- and 1.6-fold lower levels of ILA and PLA, respectively, whereas it synthesized a 1.2-fold increase in 4-OH-PLA compared to the WT strain. Thus, under the culture conditions tested, Blon_1092 was not essential for the synthesis of aromatic lactic acids. A recent study indicated that the aminotransferase Bbr_0988 from *B. breve* UCC2003 (a homolog of Blon_1439 from *B. infantis* JCM 1222^T^) is involved in the deamination of tryptophan but not phenylalanine or tyrosine.^48^

#### Type II pullulanase of *B. adolescentis* JCM 1275^T^

**Figure 5:**
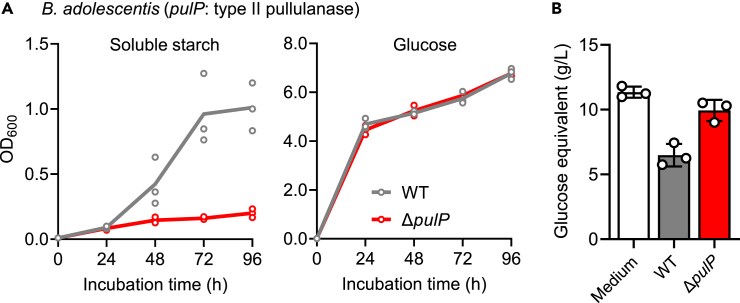
Type II pullulanase is critical for the utilization of starch in *B. adolescentis* (A) Growth of Δ*pulP* (red line) and WT (gray line) *B. adolescentis* on 1% soluble starch or glucose. Data are represented as dot plots with the mean of biological triplicates. (B) Glucose equivalent remaining in the spent medium after 96-h culture of Δ*pulP* (red bar) and WT (gray bar). Medium containing 1% soluble starch (open bar) was used as a control. Data are represented as dot plots with the mean ± SD of biological triplicates. See also Figures S3 and S5–S7.

*B. adolescentis* is generally known as a starch utilizer.^13^ Kim et al. showed that a type II pullulanase PulP of *B. adolescentis* P2P3, which possesses both GH13_32 α-amylase domain and GH 13_14 type I pullulanase domain in a polypeptide, hydrolyzes various α-glucans, such as amylose, amylopectin, soluble starch, and pullulan.^40^ The Δ*pulP* strain of *B. adolescentis* JCM 1275^T^ showed a markedly retarded growth on soluble starch from potato when compared with WT strain, whereas the growth on glucose was indistinguishable between the mutant and WT strains (Figure 5A). Thus, PulP may be the primary enzyme responsible for the assimilation of soluble starch among 13 GH13 homologues present in the genome of the strain. The total sugar concentration (glucose equivalents) after 96 h of cultivation was significantly lower in the spent medium of the WT than in the non-inoculated control and mutant (Figure 5B). Slight decrease in the total sugar concentration was observed for the mutant when compared to non-inoculated control, which might correspond to a marginal growth of the mutant on starch (OD_600_ ≈ 0.2).

#### FL2-I of *B. pseudocatenulatum* JCM 1200^T^ and FL2-III of *B. kashiwanohense* JCM 15439^T^

**Figure 6:**
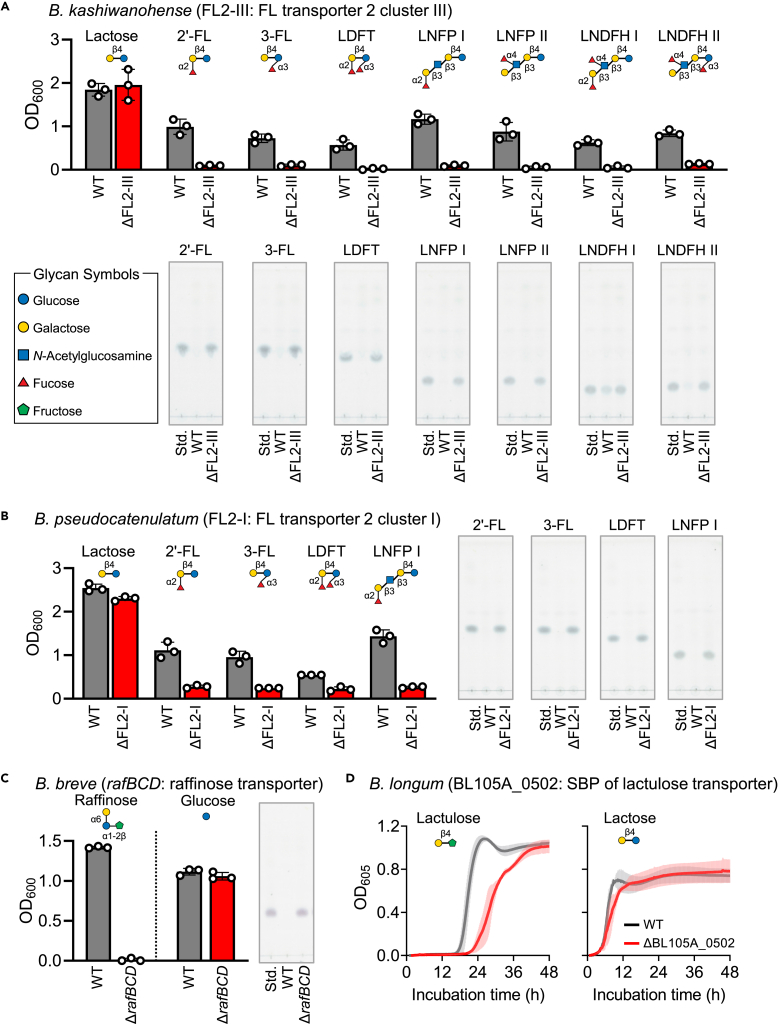
Sugar uptake ABC systems are responsible for the growth of bifidobacteria in the presence of substrates corresponding to the respective transporters (A and B) Growth of ΔFL2-III (red bar) and WT (gray bar) *B. kashiwanohense* (A) on 0.5% lactose or fucosylated HMO molecules. Growth of ΔFL2-I (red bar) and WT (gray bar) *B. pseudocatenulatum* was also examined (B). OD_600_ values after 24-h (*B. kashiwanohense*) and 48-h (*B. pseudocatenulatum*) culture are represented as dot plots with the mean ± SD of biological triplicates. Culture supernatants were used for TLC analysis, and the data are representative of biological triplicates. Contrast and brightness were adjusted to make the image clearer. (C) Growth of Δ*rafBCD* (red bar) and WT (gray bar) *B. breve* on 1% raffinose or glucose. OD_600_ values after 24 h of culture are represented as dot plots with the mean ± SD of biological triplicates. Culture supernatants grown on raffinose were used for TLC analysis, and the data are representative of three biological replicates. Contrast and brightness were adjusted to make the image clearer. (D) Growth of ΔBL105A_0502 (red line) and WT (gray line) *B. longum* on 1% lactulose or lactose. Growth was monitored by the continuous measurement of OD_605_ using the Byonoy Absorbance 96 Plate Reader (Byonoy GmbH). Data are represented as the mean ± SD of biological triplicates. See also Figures S3 and S5–S8.

ATP-binding cassette (ABC) transporters for fucosyllactose have been classified into four clades based on the signature sequences of the SBPs, i.e., FL-1 (IV), FL-2 (I), FL-2 (II), and FL-2 (III).^41^ *B. kashiwanohense* and *B. pseudocatenulatum* contain FL-2 (III) and FL-2 (I) transporters, respectively. In a previous study, we examined the substrate specificities of the two transporters by analyzing the HMO consumption behavior of WT strains and/or a recombinant *B. longum* strain heterologously expressing the transporter.^41^ In this study, by disrupting the genes in the original strains, we evaluated the physiological importance of the transporters. Only the fucosylated HMO species whose consumption was observed in the WT and/or heterologously expressing strains were tested in the growth experiments. *B. kashiwanohense* ΔFL2-III strain completely lost the ability to assimilate 2′-fucosyllactose (2′-FL), 3-FL, lactodifucotetraose (LDFT), lacto-*N*-fucopentaose I (LNFP I), LNFP II, lacto-*N*-difucohexaose I (LNDFH I), and LNDFH II, whereas the WT strain consumed them to proliferate as judged from the results of thin-layer chromatography (TLC) analysis (Figure 6A). As for *B. pseudocatenulatum* ΔFL2-I mutant, a marginal growth (OD_600_ ≈ 0.2) was detected in the medium supplemented with 2′-FL, 3-FL, LDFT, or LNFP I at the similar extent, but we did not see the difference in the intensity of sugar spots between non-inoculated control and the mutant on the TLC plate (Figure 6B). Trivial growth of *B. pseudocatenulatum* was observed when a sugar-free medium was used for cultivation (Figure S8). These results indicated that the mutant lost the ability to internalize the fucosylated HMO species.

#### rafBCD of *B. breve JCM 1192*^*T*^

Raffinose uptake by *B. breve* is mediated by the ABC transporter RafBCD. O’Connell et al. showed that a *rafB* insertional mutant of *B. breve* UCC2003 grows marginally (OD_600_ ≈ 0.3) on raffinose.^42^ The Δ*rafBCD* strain of *B. breve* JCM 1192^T^ constructed in this study showed a complete loss of the growth ability on raffinose after 24-h cultivation but showed comparable growth on glucose (Figure 6C). Raffinose remained unconsumed in the culture supernatant of the mutant strain, whereas the sugar was not detected in the supernatant of the WT strain.

#### BL105A_0502 of *B. longum* JCM 31944

Lactulose is a well-known prebiotic supplemented to formula milk.^49^^,^^50^ In a previous study, we inactivated BL105A_0502, which encodes the SBP of an ABC transporter, by suicide-vector-mediated single crossover recombination.^43^ The mutant showed delayed growth on lactulose compared with the WT strain, demonstrating the involvement of this gene in lactulose uptake. However, we were unsure whether the delayed growth was attributable to another lactulose transporter in the strain or to the reversion of the mutant to the WT strain because of plasmid excision during growth in the absence of antibiotic pressure. The scarless deletion mutant constructed in this study, ΔBL105A_0502, also showed delayed growth on lactulose, but not on lactose, when compared to the WT strain (Figure 6D).^43^ These results indicate the presence of other lactulose transporter(s) in *B. longum* JCM 31944.

## Discussion

Previous pioneering studies have developed several gene disruption systems, including single-crossover-mediated insertional mutagenesis, double-crossover-mediated gene deletion, and transposon-mediated random mutagenesis systems.^20^^,^^22^^,^^23^^,^^24^^,^^26^^,^^27^^,^^28^^,^^29^^,^^31^ Recent studies have established CRISPR/Cas-based genome-editing systems for *Bifidobacterium animalis* subsp. *lactis*^30^^,^^32^ and *B. breve*.^25^ However, each of those systems only works for specific strains or (sub)species of *Bifidobacterium*. Here, by improving the temperature sensitivity of *E. coli-Bifidobacterium* shuttle vectors, we successfully constructed an efficient scarless gene-knockout system. When using a double-crossover recombination system for gene disruption, the efficiency of obtaining FCO recombinant strains was a key step, as this step largely depends on the Ts phenotype exerted by the plasmid. In the present study, using the improved Ts plasmids and GAM, we demonstrated that FCO recombinant strains of *B. longum* were efficiently obtained at 38.5°C and 40°C (Figure 3A), temperatures generally not lethal to bifidobacteria. The plasmid was applicable not only to *B. longum* but also to other *Bifidobacterium* species (Table 1; Figure S6), including *B. infantis*, *B. breve*, *B. kashiwanohense*, *B. adolescentis*, and *B. pseudocatenulatum*. The current study represents an important advancement in that targeted gene inactivation was achieved in different (sub)species and the physiological functions of the respective genes were elucidated through the phenotypic analysis of the mutants (Figures 4, 5, and 6). It should be mentioned that neither reversion to WT nor polar effects in operons are theoretically observed in the scarless deletion mutants.

Although deletion mutants were eventually obtained, the efficiency with which SCO recombinant strains with the desired mutations (not reverting to the WT) were obtained was not high, with the exception of *B. pseudocatenulatum* (Table 1). This could be attributed to the lack of the homologous recombination pathway RecBCD in most bifidobacterial strains,^51^^,^^52^^,^^53^ which is a common issue encountered when using methods involving double-crossover-recombination-mediated gene disruption systems.^26^^,^^54^ The following are three plausible approaches that could be adopted to resolve the problem of low SCO efficiency. The first involves using counter-selection markers that facilitate the selection of SCO recombinant strains among an FCO and SCO recombinant strain pool. At present, the *pyrE* gene is the only counter-selection marker used for *Bifidobacterium* but is only available in an uracil auxotrophic Δ*pyrE* background.^55^ In other bacterial species, however, several counter-selection markers applicable to the wild-type, such as a mutated *pheS* gene encoding the α-subunit of phenylalanyl-tRNA synthetase,^56^^,^^57^^,^^58^ the *mazF* gene encoding mRNA interferase,^59^^,^^60^^,^^61^ and the *sacB* gene encoding levansucrase,^36^^,^^62^ have been used. Among these, the *pheS* and *mazF* genes have been particularly widely applied in different bacterial species,^56^^,^^57^^,^^58^^,^^59^^,^^60^^,^^61^ thereby indicating their potential application to bifidobacteria. The second approach that could be used to resolve the low SCO efficiency entails lengthening the homologous regions used for a double-crossover event. Indeed, it has previously been reported that extending the homologous region from 1 kb to 2 kb led to a 3.5-fold increase in recombination frequency (1.3 × 10^−3^–4.6 × 10^−3^) in *B. longum*.^28^ The third approach involves enhancing the expression of a replication initiation protein via modification of the promoter region and/or codons. As has previously been demonstrated,^24^ the efficiency of SCO events is influenced by the expression of the replication initiation protein and subsequent replication of an integrated plasmid from a chromosome.

Although the aforementioned problems relating to SCO events do not apply to one-step double-crossover-mediated gene deletion methods using phage-derived recombination systems such as the λ Red and RecET systems,^63^^,^^64^^,^^65^ such systems are typically only effective when applied to small groups of phylogenetically related species^66^^,^^67^ and have not been reported in bifidobacteria. Recently, the application of CRISPR/Cas systems to bifidobacteria has facilitated the induction of one-step double-crossover recombination, which leads to efficient gene deletion via a simple procedure, but appears to be effective only for certain species and strains of *Bifidobacterium*.^25^^,^^30^^,^^32^ Contrastingly, although the low efficiency of the SCO recombination assessed in the present study needs to be further resolved, our two-step double-crossover-mediated gene deletion system is applicable to different *Bifidobacterium* species. Accordingly, the Ts plasmids developed in this study can serve as fundamental tools for scarless gene deletion to elucidate the species- and strain-dependent mechanisms underlying *Bifidobacterium*-host symbioses.

### Limitations of the study

As mentioned earlier, a major limitation of the system developed in this study is that the efficiency with which SCO recombinant strains are obtained is relatively poor. One of the simplest approaches for resolving this problem is the use of counter-selection markers. However, although the use of *pyrE* as a counter-selection marker has been reported by Sakaguchi et al.,^55^ this system is currently only available for *B. longum* JCM 31944. In addition, application of the *pyrE* gene is limited to an uracil auxotrophic Δ*pyrE* background and would thus not be applicable to a wild-type background. Accordingly, further development of counter-selection marker systems will be desirable. We also note that the genus *Bifidobacterium*, which colonizes not only the human gut but also that of non-human animals, comprises more than 100 (sub)species. Consequently, further verification of the genetic tools developed in this study is required to determine whether the gene knockout system is applicable to a larger number of *Bifidobacteriu*m species.

## Resource availability

### Lead contact

Further information and requests for resources and reagents should be directed to and will be fulfilled by the lead contact, Mikiyasu Sakanaka (sakanaka.mikiyasu.5e@kyoto-u.ac.jp).

### Materials availability

Strains and vectors generated in this study are available upon reasonable request. This study did not generate other new, unique materials.

### Data and code availability

• All data reported in this paper will be shared by the lead contact upon request.
• The nucleotide sequence of pMSK197 has been deposited in the DDBJ/EMBL/GenBank databases under accession no. LC835036.
• This paper does not report original codes.
• Any additional information required to reanalyze the data reported in this paper is available from the lead contact upon request.

## Acknowledgments

This study was partly supported by 10.13039/501100001691JSPS-KAKENHI (21K14770 and 23K27128 to M.S. and 22J01706 to T.Kozakai), World Premier International Research Center Initiative (WPI); 10.13039/501100001700MEXT, Japan (to T.F. and K.M.), 10.13039/100019269Morinaga Milk Industry Co. Ltd., and North Campus Instrumental Analysis Station, 10.13039/501100005683Kyoto University. We would like to thank DSM (Heerlen, Netherlands) for providing 2′-FL, 3-FL, LDFT, LNFP I, LNFP II, and LNDFH I, Yasunobu Kano (Kyoto Pharmaceutical University, Japan) for providing pKKT427, Takuma Sakurai and Ryuta Murakami (Morinaga Milk Co., Ltd., Japan) for helpful suggestions for liquid chromatograph-tandem mass spectrometry (LC-MS/MS) analysis, and Editage (www.editage.jp) and Miriam N. Ojima for English language editing.

## Author contributions

T.Katayama. and M.S. conceived and designed the project. T.Kozakai. and M.S. constructed the Ts plasmids. T.Kozakai., T.S., and T.Katayama. generated the mutants and analyzed their phenotypes. T.Kozakai. and YS performed sugar analysis. T.Kozakai., A.N., T.O., T.Katoh., J.-z.X., and M.S. contributed to LC-MS/MS analysis. K.M. and T.F. performed AFM analysis. T.Kozakai., K.M., and M.S. drafted the manuscript. T.Katayama. and M.S. edited the manuscript. All authors discussed the data and contributed to completing the manuscript.

## Declaration of interests

Employment of T.Kozakai. (from April, 2021 to March, 2022) and M.S. (since October, 2020) at Kyoto University was in part supported by Morinaga Milk Industry Co., Ltd. T.O. and J.-z.X. are employees of Morinaga Milk Industry Co., Ltd. The authors declare no other conflicts of interest.

## Declaration of generative AI and AI-assisted technologies in the writing process

During the preparation of this work, the authors used DeepL Translate (DeepL SE, Cologne, Germany), DeepL Write (DeepL SE), and Grammarly (San Francisco, CA, USA) in order to improve language and readability. After using this tool/service, the authors reviewed and edited the content as needed and take full responsibility for the content of the publication.

## STAR★Methods

### Key resources table

**Table:** 

REAGENT or RESOURCE	SOURCE	IDENTIFIER
**Bacterial and virus strains**		

*Bifidobacterium longum* subsp. *longum* JCM 31944 (105-A)	RIKEN BRC	JCM 31944
*Bifidobacterium longum* subsp. *infantis* JCM 1222^T^	RIKEN BRC	JCM 1222
*Bifidobacterium adolescentis* JCM 1275^T^	RIKEN BRC	JCM 1275
*Bifidobacterium catenulatum* subsp. *kashiwanohense* JCM 15439^T^	RIKEN BRC	JCM 15439
*Bifidobacterium pseudocatenulatum* JCM 1200^T^	RIKEN BRC	JCM 1200
*Bifidobacterium breve* JCM 1192^T^	RIKEN BRC	JCM 1192
*B. longum* subsp. *longum* JCM 31944 *cpsD*::pMSK183-Δ*cpsD*	This paper	N/A
*B. longum* subsp. *longum* JCM 31944 *cpsD*::pMSK197-Δ*cpsD*	This paper	N/A
*B. longum* subsp. *longum* JCM 31944 *cpsD*::pKO403-Δ*cpsD*	This paper	N/A
*B. longum* subsp. *longum* JCM 31944 Δ*cpsD*	This paper	N/A
*B. longum* subsp. *infantis* JCM 1222^T^*aldh*::pMSK197-Δ*aldh*	This paper	N/A
*B. longum* subsp. *infantis* JCM 1222^T^ Blon_1092::pMSK197-ΔBlon_1092	This paper	N/A
*B. adolescentis* JCM 1275^T^*pulP*::pMSK197-Δ*pulP*	This paper	N/A
*B*. *catenulatum* subsp. *kashiwanohense* JCM 15439^T^ FL2-III::pMSK197-ΔFL2-III	This paper	N/A
*B*. *pseudocatenulatum* JCM 1200^T^ FL2-I::pMSK197-ΔFL2-I	This paper	N/A
*B*. *breve* JCM 1192^T^*rafBCD*::pMSK197-Δ*rafBCD*	This paper	N/A
*B*. *longum* subsp. *longum* JCM 31944 BL105A_0502::pMSK197-ΔBL105A_0502	This paper	N/A
*B. longum* subsp. *infantis* JCM 1222^T^ Δ*aldh*	This paper	N/A
*B. longum* subsp. *infantis* JCM 1222^T^ ΔBlon_1092	This paper	N/A
*B. adolescentis* JCM 1275^T^ Δ*pulP*	This paper	N/A
*B*. *catenulatum* subsp. *kashiwanohense* JCM 15439^T^ ΔFL2-III	This paper	N/A
*B*. *pseudocatenulatum* JCM 1200^T^ ΔFL2-I	This paper	N/A
*B*. *breve* JCM 1192^T^ Δ*rafBCD*	This paper	N/A
*B. longum* subsp. *longum* JCM 31944 ΔBL105A_0502	This paper	N/A
*Escherichia coli* DH5α	TaKaRa Bio	DH5α, Cat#9057

**Chemicals, peptides, and recombinant proteins**		

de Man, Rogosa, and Sharpe medium (MRS medium)	Difco Laboratories	Cat#288130
Luria–Bertani medium (LB medium)	Difco Laboratories	Cat#244620
Gifu anaerobic medium (GAM)	Shimadzu Diagnostics Corporation	Cat#05422
l-Cysteine Hydrochloride Monohydrate	Nacalai Tesque	Cat#10313-42
Sodium l-Ascorbate	Nacalai Tesque	Cat#03422-45
Lactulose	Sigma-Aldrich	Cat#L7877-25G
d-(+)-Raffinose Pentahydrate	FUJIFILM Wako Pure Chemical Corporation	Cat#184-00015
Starch, Soluble	FUJIFILM Wako Pure Chemical Corporation	Cat#191-03985
Lactose Monohydrate	FUJIFILM Wako Pure Chemical Corporation	Cat#128-00095
d-(+)-Glucose	Nacalai Tesque	Cat#16806-25
2′-Fucosyllactose	DSM	N/A
3-Fucosyllactose	DSM	N/A
Lactodifucotetraose (LDFT)	DSM	N/A
Lacto-*N*-fucopentaose I (LNFP I)	DSM	N/A
Lacto-*N*-fucopentaose II (LNFP II)	DSM	N/A
Lacto-*N*-difucohexaose I (LNDFH I)	DSM	N/A
Lacto-*N*-difucohexaose II (LNDFH II)	ELICITYL	Cat#GLY055-90%
In-Fusion® Snap Assembly Master Mix	TaKaRa Bio	Cat#638947
PrimeSTAR® Max DNA Polymerase	TaKaRa Bio	Cat#R045A
KOD One® PCR Master Mix -Blue-	Toyobo	Cat#KMM-201
Spectinomycin dihydrochloride	LKT Laboratories	Cat#S6018
dl-Indole-3-lactic acid	Sigma-Aldrich	Cat#I5508-250MG-A
dl-4-Hydroxyphenyllactic Acid	Tokyo Chemical Industry	Cat#H0542
l-(−)-3-Phenyllactic Acid	Tokyo Chemical Industry	Cat#P1168
3-Methyl-2-oxindole	Sigma-Aldrich	Cat#493937-5G
Triammonium Citrate	Nacalai Tesque	Cat#02502-25
Citric Acid Monohydrate	Nacalai Tesque	Cat#09106-15
Sucrose	Nacalai Tesque	Cat#30404-45
Sodium Chloride (NaCl)	Nacalai Tesque	Cat#31320-05
Disodium Hydrogenphosphate Dodecahydrate (Na_2_HPO_4_·12H_2_O)	Nacalai Tesque	Cat#31723-35
Potassium Chloride (KCl)	Nacalai Tesque	Cat#28514-75
Potassium Dihydrogen Phosphate (KH_2_PO_4_)	Nacalai Tesque	Cat#28721-55
Diphenylamine	Nacalai Tesque	Cat#13726-52
Aniline	Nacalai Tesque	Cat#02916-15
Phosphoric Acid	Nacalai Tesque	Cat#27618-55
Phenol	Nacalai Tesque	Cat#26719-65
Sulfuric Acid	Nacalai Tesque	Cat#32519-24
Ammonium Acetate	Merck	Cat#73594-25G-F
Methanol	FUJIFILM Wako Pure Chemical Corporation	Cat#134-14523

**Critical commercial assays**		

Wizard Genomic DNA Purification Kit	Promega	Cat#A1120
Wizard Plus SV Minipreps DNA Purification System	Promega	Cat#A1330
Wizard SV Gel and PCR Clean-Up System	Promega	Cat#A9281
BD BBL Gram stain kits	Becton Dickinson	Cat#BD-107845

**Deposited data**		

Nucleotide sequence of pMSK197	DDBJ/EMBL/GenBank	Accession#LC835036

**Oligonucleotides**		

	See Supplementary Table for a list of oligonucleotides	

**Recombinant DNA**		

pMSK183 (S139W substitution of RepB)	This paper	N/A
pMSK197 (S139W substitution of RepB)	This paper	Accession#LC835036
pKO403 (S139I substitution of RepB)	Sakaguchi et al.^28^	N/A
pKKT427	Yasui et al.^68^	N/A
pMSK162	This paper	N/A
pMSK162-Ile (S139I substitution of RepB)	This paper	N/A
pMSK162-Phe (S139F substitution of RepB)	This paper	N/A
pMSK162-Leu (S139L substitution of RepB)	This paper	N/A
pMSK162-Val (S139V substitution of RepB)	This paper	N/A
pMSK162-Thr (S139T substitution of RepB)	This paper	N/A
pMSK162-Ala (S139A substitution of RepB)	This paper	N/A
pMSK162-Tyr (S139Y substitution of RepB)	This paper	N/A
pMSK162-His (S139H substitution of RepB)	This paper	N/A
pMSK162-Lys (S139K substitution of RepB)	This paper	N/A
pMSK162-Cys (S139C substitution of RepB)	This paper	N/A
pMSK162-Arg (S139R substitution of RepB)	This paper	N/A
pMSK162-Gly (S139G substitution of RepB)	This paper	N/A
pMSK183-Δ*cpsD*	This paper	N/A
pMSK197-Δ*cpsD*	This paper	N/A
pKO403-Δ*cpsD*	This paper	N/A
pMSK197-Δ*aldh*	This paper	N/A
pMSK197-ΔBlon_1092	This paper	N/A
pMSK197-Δ*pulP*	This paper	N/A
pMSK197-ΔFL2-III	This paper	N/A
pMSK197-ΔFL2-I	This paper	N/A
pMSK197-Δ*rafBCD*	This paper	N/A
pMSK197-ΔBL105A_0502	This paper	N/A

**Software and algorithms**		

SnapGene® Viewer 7.0.1	GSL Biotech	N/A
JPK Data Processing software Version 7.0.165	Bruker Nano GmbH	N/A

### Experimental model and study participant details

#### Bacterial strains, growth media, and culture conditions

Information pertaining to the *Bifidobacterium* wild-type and mutant strains used in this study are shown in the key resources table. *B. longum* subsp. *longum* JCM 31944 (105-A), *B. longum* subsp. *infantis* JCM 1222^T^, *B. adolescentis* JCM 1275^T^, *B*. *catenulatum* subsp. *kashiwanohense* JCM 15439^T^, *B. pseudocatenulatum* JCM 1200^T^, and *B. breve* JCM 1192^T^ were from the RIKEN BRC through the National BioResource Project of the MEXT, Japan. The bifidobacteria were grown anoxically using an AnaeroPack culture system (Mitsubishi Gas Chemical, Tokyo, Japan) at the indicated temperatures (30, 37, 38.5, or 40°C). GAM (Shimadzu Diagnostics Corporation, Tokyo, Japan) or MRS-CS medium was used for cultivation. When examining sugar utilization capacity, sugar was added to the MRS-CS medium at a final concentration of 0.5 or 1.0% (w/v) and cultivation was performed at 37°C. *Escherichia coli* DH5α, which was used as a host for genetic manipulation, was grown at 30 or 37°C in LB medium. When necessary, Sp was added to the medium at the final concentrations of 30 μg/mL for GAM, 75 μg/mL for MRS-CS, and 60 μg/mL for LB. Unless otherwise indicated, growth was monitored by measuring optical density at 600 nm (OD_600_).

### Method details

#### DNA manipulation

Primers and template DNAs used in this study are listed in Table S1. The primers (oligonucleotides) were synthesized commercially by Eurofins Genomics K.K (Tokyo, Japan). High-fidelity PCR was performed using PrimeSTAR Max DNA polymerase (TaKaRa Bio, Shiga, Japan) and purified genomic DNA as a template. For colony PCR, the KOD One polymerase (TOYOBO, Shiga, Japan) was used. The genomic DNA of *Bifidobacterium* was extracted using the Wizard Genomic DNA Purification Kit (Promega, Madison, WI, USA). Plasmid DNA was extracted using the Wizard Plus SV Minipreps DNA Purification System (Promega), and PCR products were extracted using the Wizard SV Gel and PCR Clean-up System (Promega). DNA ligation was conducted using In-Fusion Snap Assembly Master Mix (TaKaRa Bio). Sanger sequencing was performed by Eurofins Genomics K.K (Tokyo, Japan).

#### Transformation of bifidobacteria

Electrotransformation of bifidobacteria was performed according to the method described by Sakanaka et al. (22), with slight modifications. Bifidobacteria were cultured in liquid GAM until reaching an OD_600_ of between 0.4 and 0.6. Three-milliliter volumes of cultured cells were harvested by centrifugation (5,000 × *g*, 3 min, 4°C), washed twice with 3 mL of 1 mM ammonium citrate buffer (pH 6.0; triammonium citrate–citric acid) containing 50 mM sucrose, and concentrated to 150 μL using the same buffer. Aliquots (50 μL) of cells were then electroporated with 0.1–1.5 μg of plasmid DNA using a Gene Pulser Xcell Electroporation System (Bio-Rad Laboratories, Hercules, CA, USA) with settings of 10 kV/cm, 25 μF, and 200 Ω (0.2-cm cuvette). The pulsed cells were immediately mixed with 1 mL of liquid GAM, incubated anoxically at 30°C for 3 h, and then spread onto GAM-Sp agar plates. The plates were incubated anoxically at 30°C for 3–4 days to obtain transformant colonies.

#### Construction of the pMSK162 shuttle vector

A 3.0-kb *E. coli*–*Bifidobacterium* shuttle vector pMSK162, which is an *sso* (single-strand origin)-eliminated version of pMSK187 (3.2 kb),^69^^,^^70^ was constructed by ligating pTB6 replicon (Δ*sso*), pUC replicon, and Sp^R^ gene that were amplified from pKKT427.^68^ The primers used are listed in Table S1 (Nos. 1–3).

#### Saturation mutagenesis at Ser-139 of RepB and evaluation of Ts phenotype

Site-directed mutagenesis was performed using a PCR-based method with pMSK162 (3.0 kb) or pKO403 (3.9 kb),^28^ as a template. The primers used are listed in Table S1 (Nos. 4–22). To examine the Ts phenotype exhibited by the mutated and parental plasmids, *B. longum* JCM 31944 was used as the host. The strain was also used with the parental plasmid of pKO403, pKKT427 bearing WT *repB*.^68^ The plasmid-carrying colonies that appeared on GAM-Sp agar plate incubated at 30°C were picked up and suspended in 0.85% (w/v) NaCl. The suspensions were serially diluted, spotted onto both GAM and GAM-Sp agar plates, and incubated at permissive (30°C) and non-permissive (38.5°C) temperatures. CFU on plates incubated at different temperatures were compared to estimate plasmid stability at the indicated temperatures.

#### Scarless gene deletion in *Bifidobacterium* species

Ts plasmid-based scarless gene deletion was performed using a two-step double-crossover recombination (Figure 1). The upstream and downstream 1.0-kb regions of the target gene were amplified by PCR, and the resulting DNA fragments were ligated in tandem with linearized pMSK183, pMSK197, or pKO403. Linearization of the plasmids was carried out by NsiI digestion of pMSK183 and inverse PCR for pMSK197 and pKO403. The primer pairs used are listed in Table S1 (Nos. 23–40 and 49).

Ts plasmids were individually introduced into the respective *Bifidobacterium* species by electrotransformation, as described above. Sp^R^ transformants obtained on GAM-Sp agar plates were picked up and incubated in liquid GAM-Sp at 30°C for two days (Figure 1B(i)). The diluted culture was spread onto GAM-Sp agar plates and incubated at 38.5 or 40°C for 2–4 days to obtain the FCO recombinant strains (Figure 1B(ii)). Plasmid integration into the targeted locus on the chromosome was verified by colony or genomic PCR using the primer pairs listed in Table S1 (Nos. 41–48). The FCO recombinant strain was then inoculated into liquid MRS-CS and incubated at 30°C for 1–2 days to allow SCO recombination to occur (Figure 1B(iii)). During this process, the Ts plasmid is assumed to be cured from the cells of the SCO recombinant strains, indicating the subsequent growth does not require the incubation at 30, 38.5, or 40°C. Thus, after the appropriately diluted culture was spread onto MRS-CS agar plates, the cultivation was performed at 37°C (optimal temperature for bifidobacteria). Colonies that appeared on the agar plates were replicated on antibiotic-free and Sp-supplemented agar plates, and colonies with the Sp^S^ phenotype were selected. Candidate Sp^S^ colonies were subjected to colony PCR (Table S1, Nos. 41–48) to examine whether SCO recombination had occurred. Δ*cpsD* strains of *B. longum* JCM 31944 were visually screened as mentioned in the results section (Figure 3B). The SCO steps are shown in Figure 1B(iii) was repeated until the desired deletion mutant was obtained.

#### Microscopic analysis

Optical microscopy was performed as follows. *B. longum* Δ*cpsD* and WT were grown logarithmically in liquid MRS-CS, washed once with phosphate-buffered saline (8.1 mM Na_2_HPO_4_, 1.47 mM KH_2_PO_4_, 2.68 mM KCl, and 137 mM NaCl), and spotted onto microscope slide glass. After fire drying, India ink staining and Gram staining were conducted, for latter of which BD BBL Gram stain kits (Becton Dickinson, NJ, USA) were used. Micrographs were obtained using a BZ-X810 microscope (Keyence, Osaka, Japan).

AFM was performed using JPK ULTRA Speed 2 (Bruker Nano GmbH, Berlin, Germany) equipped with a cantilever with a nominal spring constant of 2 N/m (240AC-NG, OPUS, MikroMasch, Tallinn, Estonia) combined with an inverted optical microscope (IX, Olympus, Tokyo, Japan). The colonies grown on MRS-CS agar plates were collected with a pipette tip and attached onto a glass substrate (S1111; Matsunami Glass Ind., Ltd., Osaka, Japan), and AFM measurements were performed using QI mode of JPK-AFM in air at room temperature (25°C). The force setpoint value was 10 nN, and the measured size was 15 × 15 μm^2^ with 128 × 128 pixels. The Z length and vertical tip scanning speed were 0.3 μm and 188 μm/s for WT, and 0.9 μm and 562 μm/s for Δ*cpsD*, respectively (Figure 3F).

To visualize AFM images, JPK Data Processing software (Version 7.0.165, Bruker Nano GmbH) was used with image processing techniques, including plane fit (degree: 1), line leveling (degree: 1, pixel range of exclude high features between 0% and 50%), median filter (mask width: 3, tolerance: 3.00), low-pass filter (operation: Savitzky-Golay filter, smoothing width: 7, order: 4), bicubic interpolation. The extracellular layer of WT and Δ*cpsD* strains was visualized by adjusting the dark gold color contrast within the range of −20 to 50 nm (Figures S9A and S9B). By contrast, cell heights were visualized within the range of 0–550 nm, but in this case, the contrasts of the extracellular layers disappeared (Figures S9C and S9D). Therefore, the cell heights were visualized using three-dimensional (3D) visualization (Z ratio: 10%), and the resulting images shown in Figures S9E and S9F were combined with the images of Figures S9A (WT) and S9B (Δ*cpsD*), respectively, to visualize both extracellular layers and cell heights (Figures S9G and S9H).

#### Sugar consumption analysis

Bacterial consumption of sugars was qualitatively analyzed using thin-layer chromatography with eluent (1-butanol: acetic acid: water = 2: 1: 1), as described previously.^71^ The sugars were visualized using a diphenylamine-aniline-phosphoric acid reagent.^72^

Starch degradation was estimated by determining the total sugar concentration in culture supernatants using the phenol-sulfuric acid method.^73^ The supernatant was diluted 400-fold, with 100 μL of the diluted solution subsequently being mixed with 100 μL of 5% (v/v) phenol and 500 μL of concentrated sulfuric acid (in that order). After incubation at room temperature for 10 min, the mixture was cooled on ice for 10 min, followed by measurement of the absorbance at 490 nm using a Multiskan SkyHigh plate reader (Thermo Fisher Scientific). A standard curve was generated using known concentrations of glucose.

#### Quantification of aromatic lactic acids

An LCMS-8030 triple quadrupole mass spectrometry system (LC-MS/MS; Shimadzu, Kyoto, Japan) equipped with an XBridge BEH Phenyl column (4.6 mm × 150 mm; Waters, Milford, MA, USA) was used to quantify PHA, 4-OH-PHA, and ILA. The column temperature was maintained at 40°C. Elution was carried out at a flow rate of 0.2 mL/min by a gradient system between mobile phase A [0.1% (w/v) ammonium acetate in water] and mobile phase B [0.1% (w/v) ammonium acetate in methanol]. The gradient consisted of 2% B for 0–2 min, 2 to 30% B for 2–5 min, 30 to 35% B for 5–10 min, 35% B for 10–15 min, 35 to 99% B for 15–28 min, and 99% B for 28–33 min. Prior to injection, culture supernatants were diluted by 30-fold with mobile phase A supplemented with 0.5 μg/mL 3-methyl-2-oxindole as an internal standard. The ionization and detection methods are listed in Table S2. Known concentrations of the respective compounds were used to generate the standard curves.

### Quantification and statistical analysis

Experiments were performed in at least biological duplicates. The quantitative data in Figures 2A, 2B, 3A, 5A, S2, and S5B were represented as dot plots with mean, while those in Figures 4B, 5B, 6A–6C, and S8 were expressed as dot plots with mean ± standard deviation (SD). Data in Figures 6D and S5A were represented as mean ± SD. Data in Figure 3C were shown for each replicate. The statistical analysis was not performed in this study.
