# Supplementary material for: An improved temperature-sensitive shuttle vector system for scarless gene deletion in human-gut-associated Bifidobacterium species
Source: iScience. 2024 Oct 1;27(11):111080. doi: 10.1016/j.isci.2024.111080 (PMC11536034; doi:10.1016/j.isci.2024.111080)
Supplement: Document S1. Figures S1–S9 and Tables S1 and S2 [file mmc1.pdf]

iScience, Volume *27*

Supplemental information

An improved temperature-sensitive shuttle vector system for scarless gene deletion in human-gut-associated *Bifidobacterium* species

Tomoya Kozakai, Aruto Nakajima, Keisuke Miyazawa, Yuki Sasaki, Toshitaka Odamaki, Toshihiko Katoh, Takeshi Fukuma, Jin-zhong Xiao, Tohru Suzuki, Takane Katayama, and Mikiyasu Sakanaka

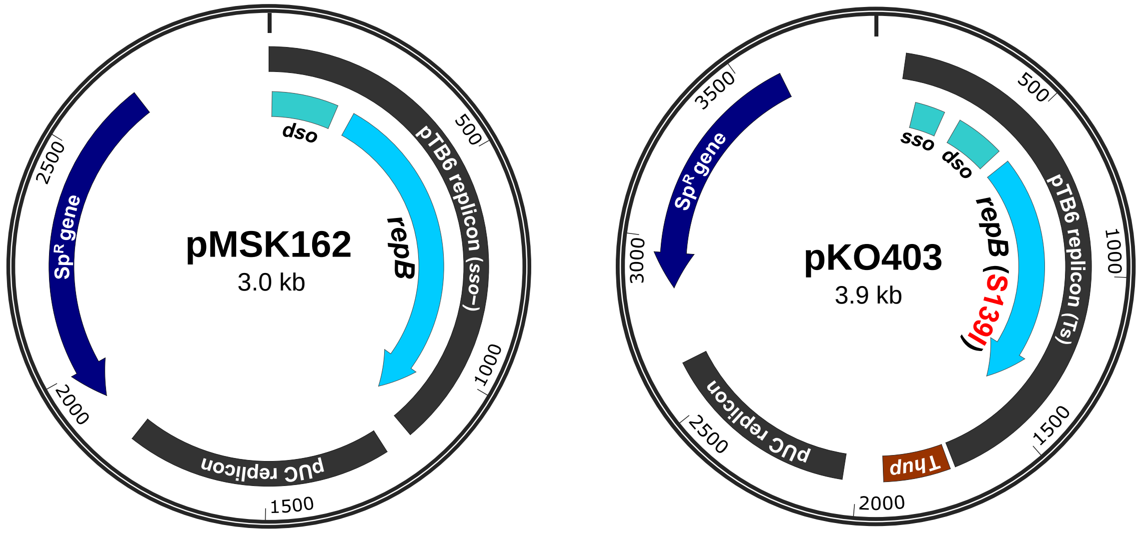

Fig. S1. Schematic representation of the *E. coli–Bifidobacterium* shuttle vectors, pMSK162 and pKO403, related to Figure 2.

pMSK162 harbors WT *repB*, whereas pKO403 harbors previously reported mutated *repB* (S139I [S1]). pTB6 replicon, replicon for bifidobacteria; *sso*, single-strand origin for replication; *dso*, double-strand origin for replication; *repB*, replication initiation protein gene; T*hup*, transcription terminator of *hup* gene from *B. longum*; pUC replicon, replicon for *E. coli*; Sp^{R} gene, spectinomycin resistance gene. Open reading frames are indicated by arrows. Plasmid maps were drawn using SnapGene Viewer 7.0.1.

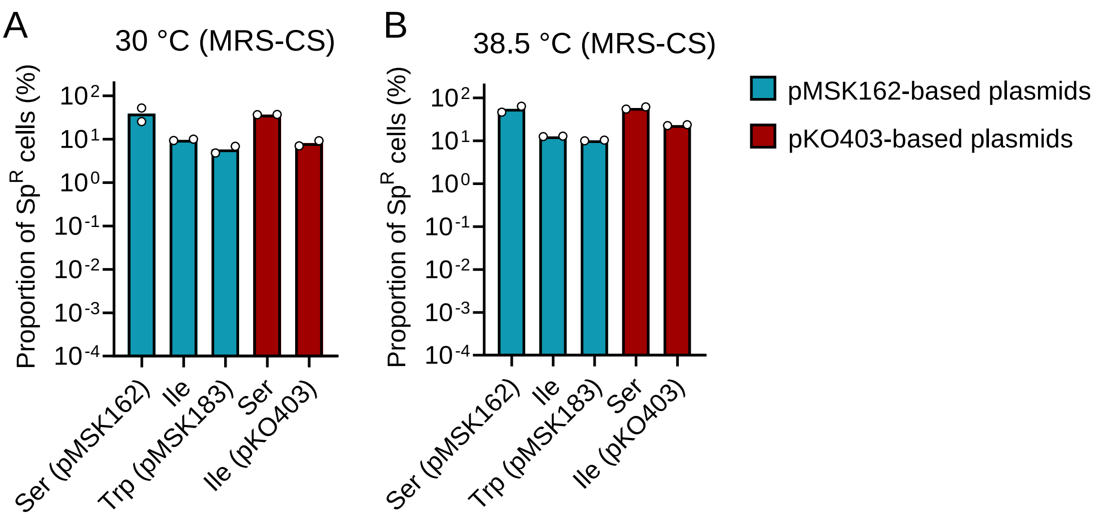

Fig. S2. MRS-CS medium is not suitable to achieve the Ts phenotype in *B. longum* JCM 31944, related to Figure 2.
(A, B) Effect of the 139th amino acid residue of RepB on plasmid stability when incubated on MRS-CS plates. The selected transformant cells (Fig. 2) were spread onto the MRS-CS and MRS-CS-Sp plates. The plates were incubated at 30 ºC (A) or 38.5 ºC (B) to determine the CFU. Plasmid stability is shown as the percentage of Sp^R cells in the total number of cells. Blue bars indicate pMSK162 or its derivative-carrying transformant cells, whereas red bars indicate pKO403 or its derivative-carrying cells. Data are represented as dot plots with mean of biological duplicates.

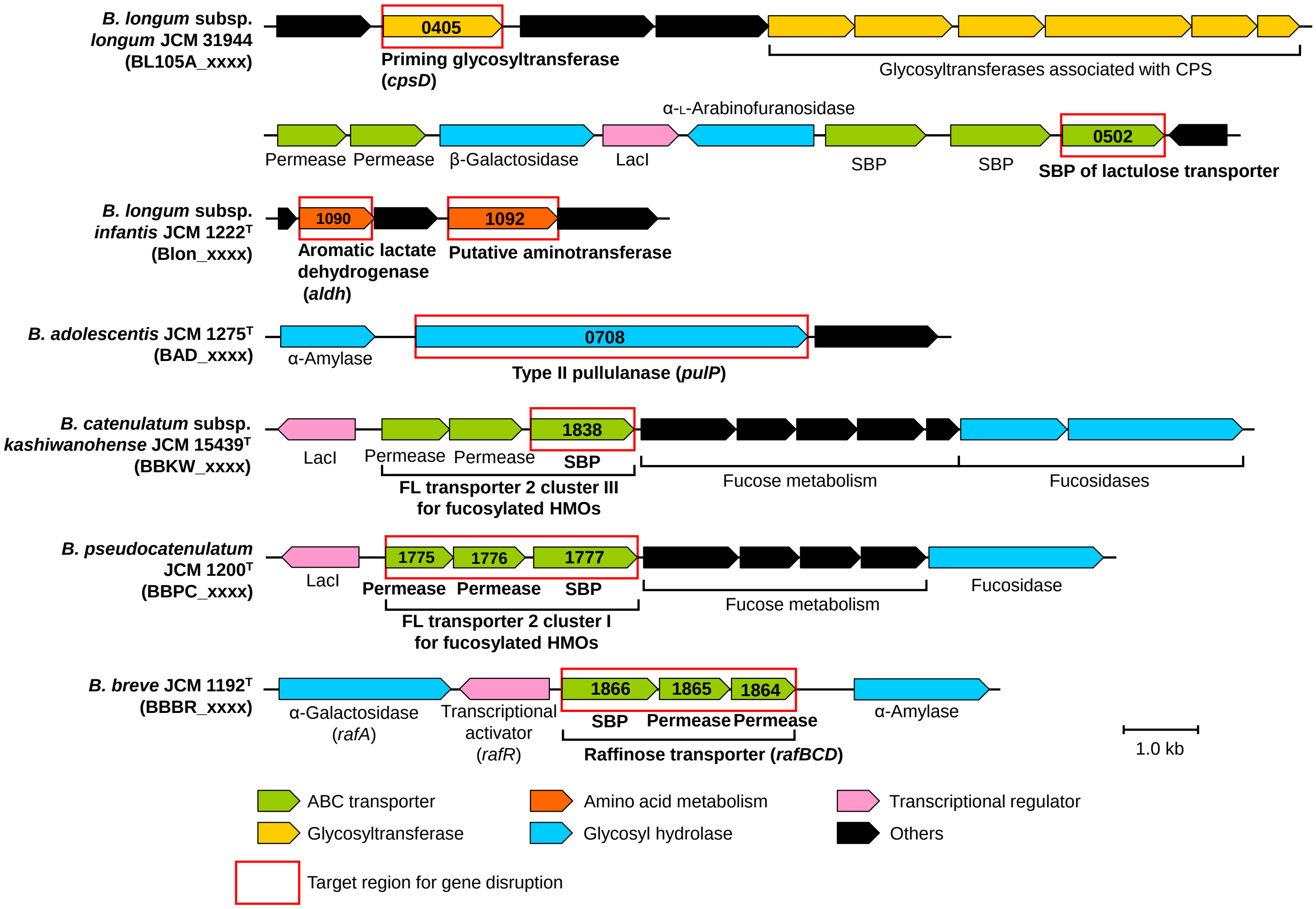

Fig. S3. Gene clusters containing the target region for gene deletion, related to Figures 3 to 6. The target gene is indicated by an open box with a red border. Gene organization was shown using drawGeneArrows3 (http://www.ige.tohoku.ac.jp/joho/labhome/tool.html).

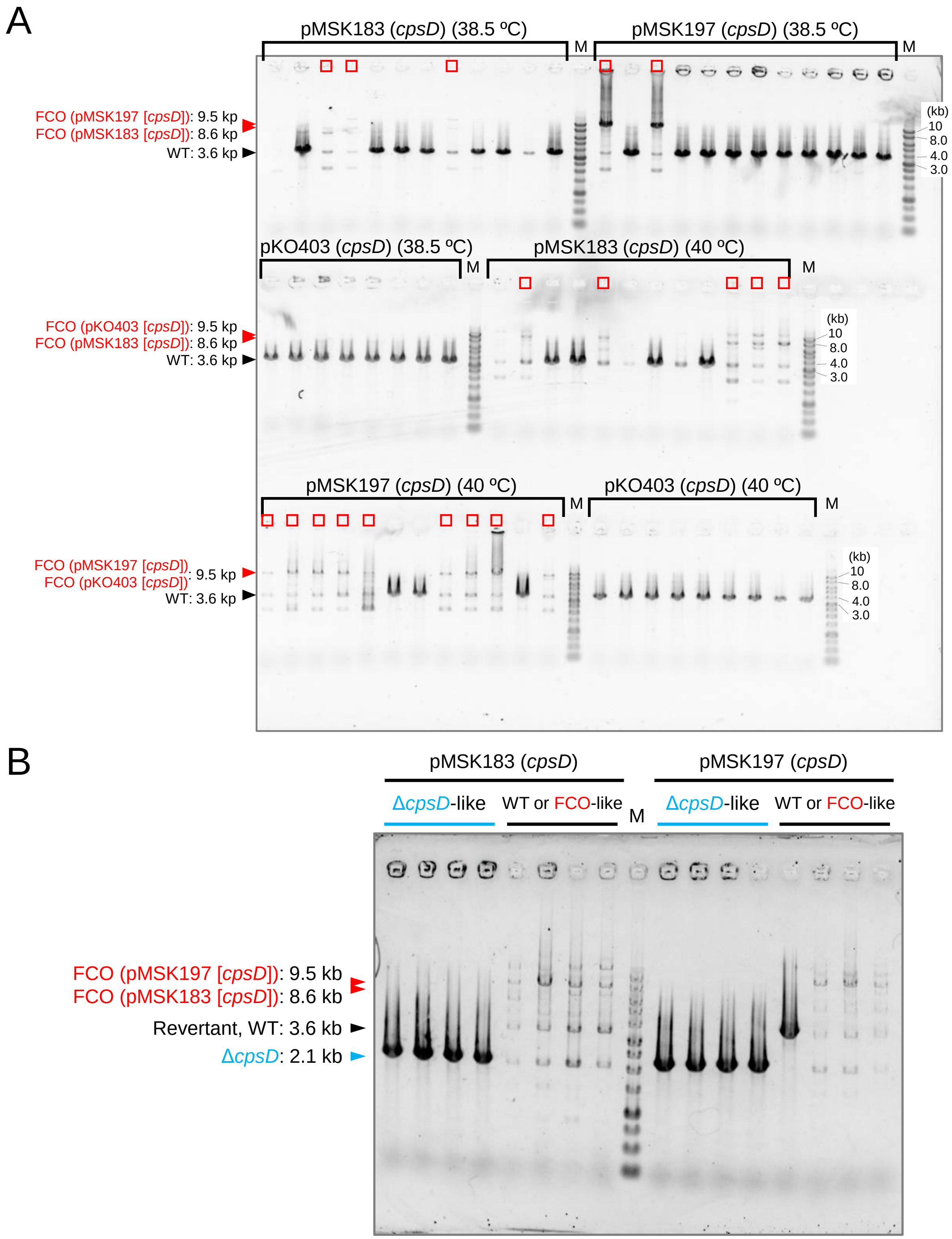

Fig. S4. First crossover (FCO) and second crossover (SCO) recombination at the *cpsD* locus were verified by colony PCR followed by agarose electrophoresis, related to Figure 3.
(A) Verification of FCO recombination. Candidate FCO recombinant strains were obtained by incubating the transformants carrying pMSK183 (*cpsD*), pMSK197 (*cpsD*), or pKO403 (*cpsD*) at non-permissive temperatures (38.5 or 40 ºC) on the GAM-Sp plate. Colony PCR was performed using a primer pair designed to anneal the outside upstream and downstream 1-kb regions of *cpsD* (Table S1 No. 41). Red and black arrows indicate the positions of PCR products from the FCO recombinant and WT strains, respectively. Red squares indicate the samples determined to be FCO recombinant strains. An Excelband 1-kbp DNA ladder (COSMO BIO) was used as a marker (M). Images were taken with iBright CL1500 Imaging System (Thermo Fischer Scientific) with default settings, and the color of the image was inverted to make it more visible. See also Fig. 1A. (B) Verification of SCO recombination. Colony PCR and agarose electrophoresis were performed as in (A). The Δ*cpsD*-like thin colonies or WT or FCO-like thick colonies (see also Fig. 3B), which were obtained after subculture of the FCO recombinant strains in antibiotic-free MRS-CS liquid medium (Fig. 1B, iii), were used as the template DNA. Blue, red, and black arrows indicate the positions of the PCR products from the Δ*cpsD*, FCO recombinant, and WT (revertant) strains, respectively.

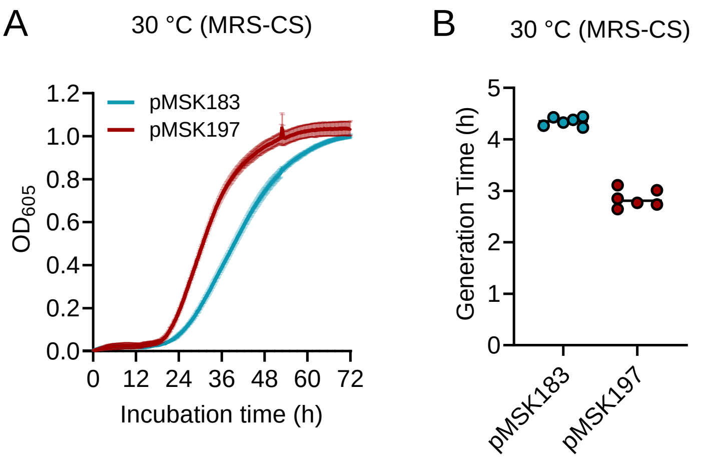

Fig. S5. pMSK197 increases the growth of *B. longum* better than pMSK183, related to Figures 4 to 6. (A) Growth of pMSK183- or pMSK197-carrying *B. longum* strains at 30 ºC in MRS-CS medium containing Sp. Growth was monitored by the continuous measurement of OD_{605} using the Byonoy Absorbance 96 Plate Reader (Byonoy GmbH). Data are represented as the mean ± SD of biological sextuplicates. (B) Generation time of pMSK183- or pMSK197-carrying *B. longum* strains. The generation time was determined using OD_{605} values of 0.1–0.2, which indicates the logarithmic growth phase. Data in (A) was used for analysis. Data are represented as dot plots with the mean of biological sextuplicates.

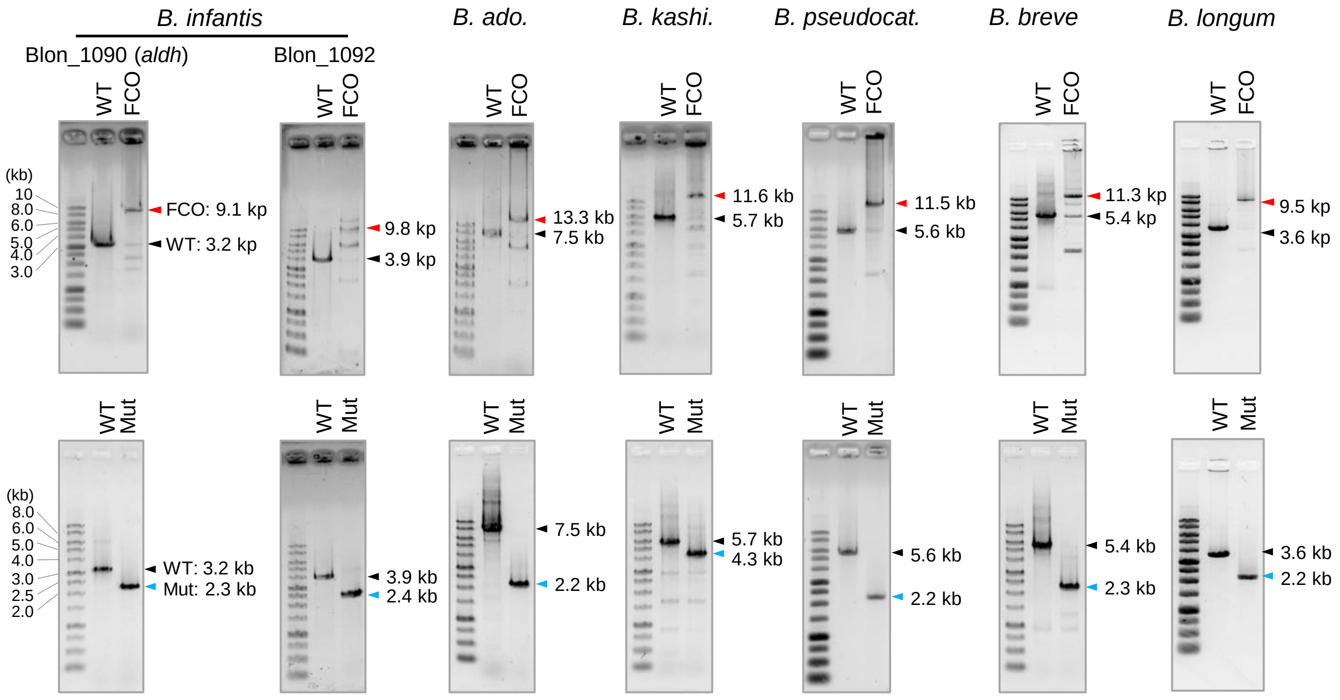

Fig. S6. First crossover (FCO) and second crossover (SCO) recombination in different *Bifidobacterium* species was verified by genomic PCR followed by agarose electrophoresis, related to Figures 4 to 6.

Genomic PCR was performed using primer pairs designed to anneal the outside upstream and downstream 1-kb regions of the target gene (Table S1, No. 42–48). WT, FCO recombinant, and SCO recombinant mutant (Mut) strains were used as templates. Upper panels show the agarose electrophoresis results for verifying FCO recombination, whereas the lower panels show the results for verifying SCO recombination. Blue, red, and black arrows indicate the positions of the PCR products from the Mut, FCO recombinant, and WT strains, respectively. Images were taken with the iBright CL1500 Imaging System (Thermo Fischer Scientific) with default settings, and the color of the image was inverted to make it more visible. See also Fig. 1A.

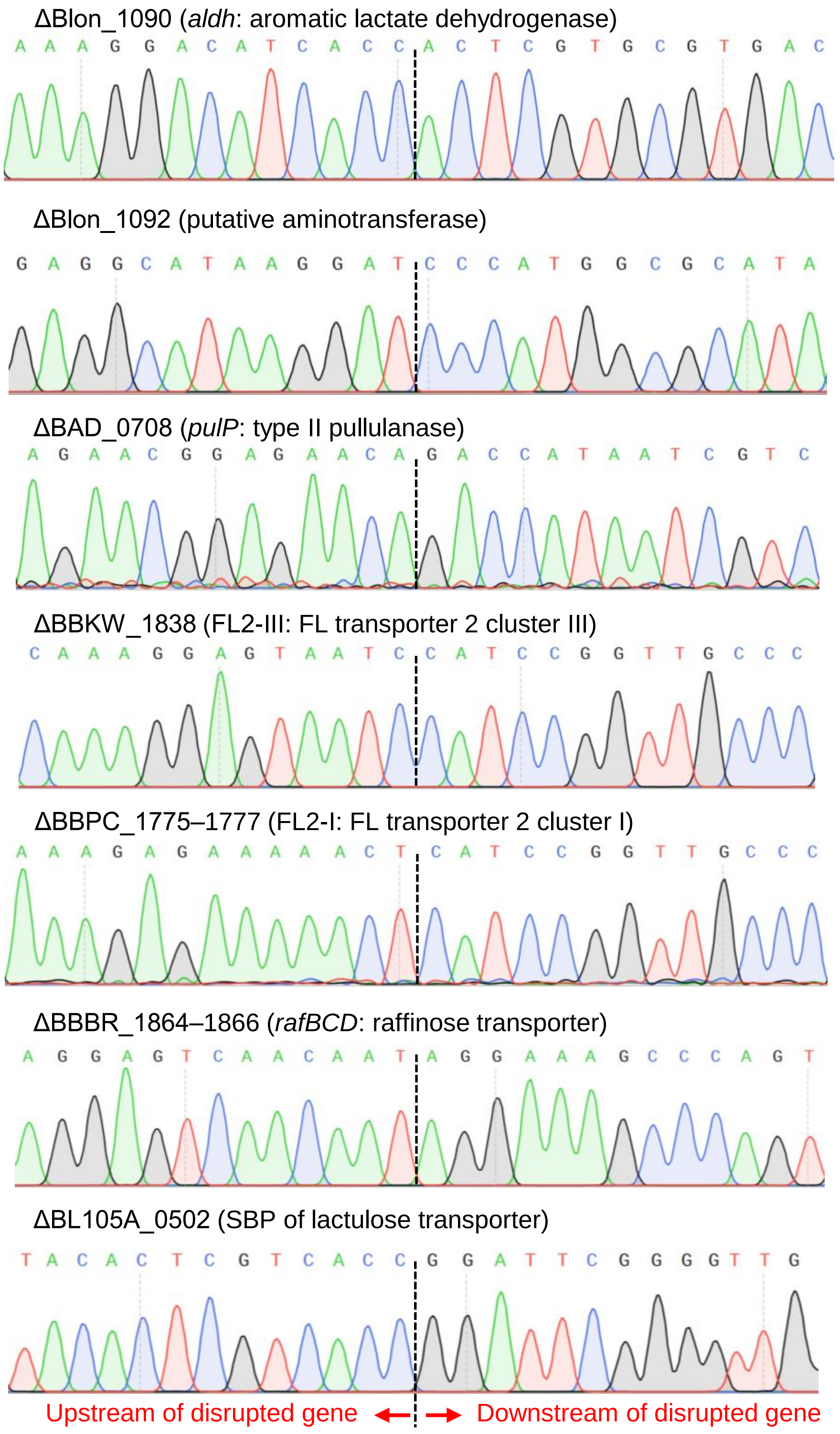

Fig. S7. Waveform data obtained by Sanger sequencing of the amplified fragments at the target loci, related to Figures 4 to 6.

Amplified fragments from the respective mutants (Fig. S6) were used for sequencing. Dotted lines indicate the boundary between the upstream and downstream regions, indicating the successful deletion of target genes.

B. pseudocatenulatum
(FL2-I: FL transporter 2 cluster I)

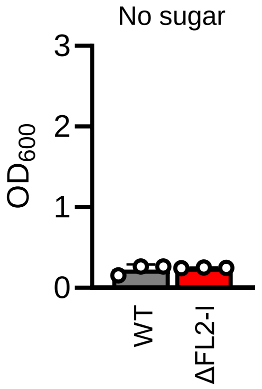

Fig. S8. Growth of *B. pseudocatenulatum* strains without a sugar source, related to Figure 6. Growth of ΔFL2-I (red bar) and WT (gray bar) *B. pseudocatenulatum* in MRS-CS medium without a sugar source. OD_{600} values after 48 h of culture are shown as dot plots with the mean ± SD of biological triplicates.

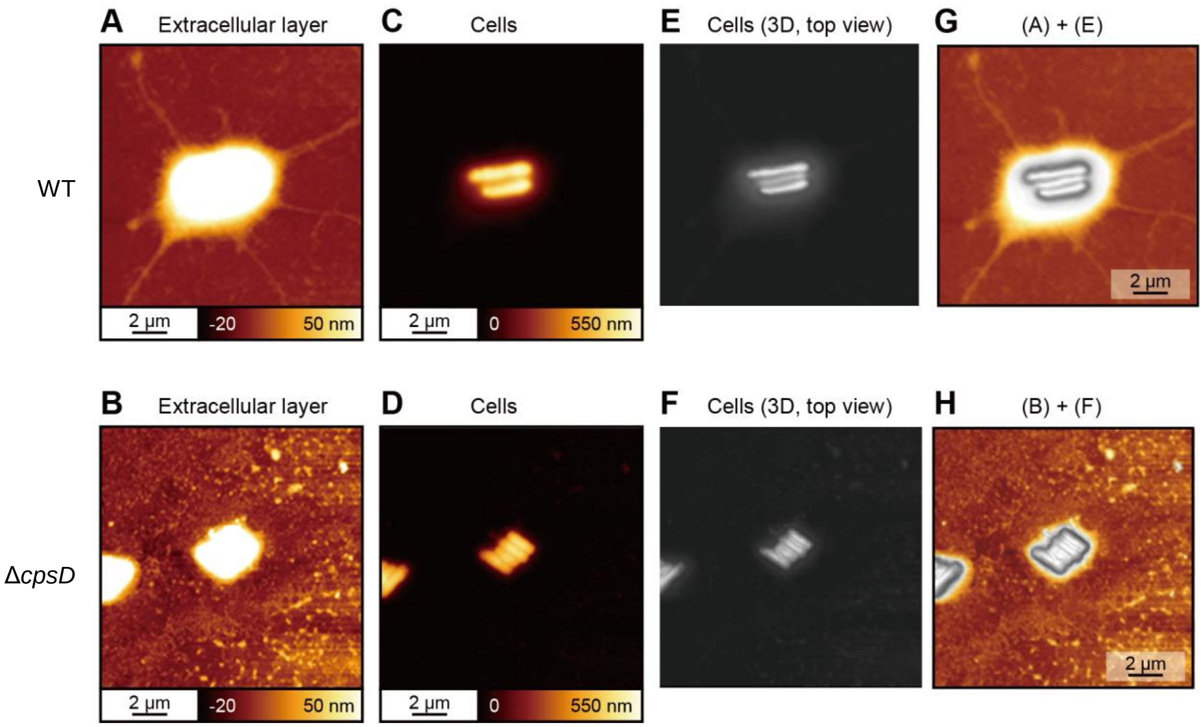

Fig. S9. Atomic force microscopy (AFM) images of WT (A, C, E, G) and Δ*cpsD* (B, D, F, H) strains, related to Figure 3 and STAR Methods.

(A–D) The dark gold color contrasts were adjusted within the range of –20 to 50 nm (A,B) and 0 to 550 nm (C,D). (E,F) Three-dimensional (3D) visualizations of the AFM images in Fig. S9C and D are shown in (E) and (F), respectively. (G,H) The AFM images in Fig. S9A and E were combined, as shown in (G). The images in Fig. S9B and F were also combined to produce the image in (H). See also STAR Methods.

Table S1. Primers used in this study, related to STAR Methods

No.	Target	Nucleotide sequence (5′ to 3′)[a]	Template[b]
1	pTB6 replicon (Δ*sso*)	atgcataatggcgcttaggcgacgtccgaaaaaaggcgctg<br>taattcatgaaacgtaaaacccccggtcagaccgaccgagggttttcagaggcgcgg acacggccgcta	pKKT427
2	pUC replicon	acgtttcatgaattaatggcgcttaggcgtttttccataggctccgccc<br>gtatgctcggtcatatgttggaacgtcccgtagaaaagatcaaaggatc	pKKT427
3	Sp^R gene	tatgaccgagcatacattaaataataaaacaaaaa<br>agcgccattatgcataattcatgaaacgttttcgttcgtgaatacatgt	pKKT427
4	Substitution at the position 139 of RepB with Ile	gttcgccgacctcaagATCaagtacgccaaggagt<br>actccttggcgtacttGATcttgaggtcggcgaac	pMSK162
5	Substitution at the position 139 of RepB with Phe	gttcgccgacctcaagTTCaagtacgccaaggagt<br>actccttggcgtacttGAActtgaggtcggcgaac	pMSK162
6	Substitution at the position 139 of RepB with Leu	gttcgccgacctcaagCTCaagtacgccaaggagt<br>actccttggcgtacttGAGcttgaggtcggcgaac	pMSK162
7	Substitution at the position 139 of RepB with Met	gttcgccgacctcaagATGaagtacgccaaggagt<br>actccttggcgtacttCATcttgaggtcggcgaac	pMSK162
8	Substitution at the position 139 of RepB with Val	gttcgccgacctcaagGTCaagtacgccaaggagt<br>actccttggcgtacttGACcttgaggtcggcgaac	pMSK162
9	Substitution at the position 139 of RepB with Pro	gttcgccgacctcaagCCCaagtacgccaaggagt<br>actccttggcgtacttGGGcttgaggtcggcgaac	pMSK162
10	Substitution at the position 139 of RepB with Thr	gttcgccgacctcaagACCaagtacgccaaggagt<br>actccttggcgtacttGGTcttgaggtcggcgaac	pMSK162
11	Substitution at the position 139 of RepB with Ala	gttcgccgacctcaagGCCaagtacgccaaggagt<br>actccttggcgtacttGGCcttgaggtcggcgaac	pMSK162
12	Substitution at the position 139 of RepB with Tyr	gttcgccgacctcaagTACaagtacgccaaggagt<br>actccttggcgtacttGTActtgaggtcggcgaac	pMSK162
13	Substitution at the position 139 of RepB with His	gttcgccgacctcaagCACaagtacgccaaggagt<br>actccttggcgtacttGTGcttgaggtcggcgaac	pMSK162
14	Substitution at the position 139 of RepB with Gln	gttcgccgacctcaagCAGaagtacgccaaggagt<br>actccttggcgtacttCTGcttgaggtcggcgaac	pMSK162
15	Substitution at the position 139 of RepB with Asn	gttcgccgacctcaagAACaagtacgccaaggagt<br>actccttggcgtacttGTTcttgaggtcggcgaac	pMSK162
16	Substitution at the position 139 of RepB with Lys	gttcgccgacctcaagAAGaagtacgccaaggagt<br>actccttggcgtacttCTTcttgaggtcggcgaac	pMSK162
17	Substitution at the position 139 of RepB with Asp	gttcgccgacctcaagGACaagtacgccaaggagt<br>actccttggcgtacttGTCcttgaggtcggcgaac	pMSK162
18	Substitution at the position 139 of RepB with Glu	gttcgccgacctcaagGAGaagtacgccaaggagt<br>actccttggcgtacttCTCcttgaggtcggcgaac	pMSK162
19	Substitution at the position 139 of RepB with Cys	gttcgccgacctcaagTGCaagtacgccaaggagt<br>actccttggcgtacttGCActtgaggtcggcgaac	pMSK162
20	Substitution at the position 139 of RepB with Trp	gttcgccgacctcaagTGGaagtacgccaaggagt<br>actccttggcgtacttCCActtgaggtcggcgaac	pMSK162 and pKO403
21	Substitution at the position 139 of RepB with Arg	gttcgccgacctcaagCGCaagtacgccaaggagt<br>actccttggcgtacttGCGcttgaggtcggcgaac	pMSK162
22	Substitution at the position 139 of RepB with Gly	gttcgccgacctcaagGGCaagtacgccaaggagt<br>actccttggcgtacttGCCcttgaggtcggcgaac	pMSK162
23	Upstream region of *cpsD* (for pMSK183)	ggacgttccaacataggctgaggctggacttccc<br>cgaaacgggaaggaagtggctgagctgcttttggc	*B. longum* JCM 31944 genome (WT)
24	Downstream region of *cpsD* (for pMSK183)	ttccttcccgtttcgctgtcac<br>gtatgctcggtcatatggcccacgcagttggaatg	*B. longum* JCM 31944 genome (WT)
25	Upstream region of *cpsD* (for pMSK197 and pKO403)	gtatatatgagtactggctgaggctggacttccc<br>cgaaacgggaaggaagtggctgagctgcttttggc	*B. longum* JCM 31944 genome (WT)
26	Downstream region of *cpsD* (for pMSK197 and pKO403)	ttccttcccgtttcgctgtcac<br>atttaatgggaattctggcccacgcagttggaatg	*B. longum* JCM 31944 genome (WT)
27	Upstream region of BL105A_0502 (for pMSK197)	gtatatatgagtacttacaacaaggaggtcttcga<br>ggtgacgagtgtactaggcg	*B. longum* JCM 31944 genome (WT)
28	Downstream region of BL105A_0502 (for pMSK197)	agtacactcgtcaccggattcggggttgggtcg<br>atttaatgggaattctgtggttgcgccgtcttc	*B. longum* JCM 31944 genome (WT)
29	Upstream region of *rafBCD* (for pMSK197)	gtatatatgagtactcccactggtgcagcgc<br>attgttgactcctttatctttttccttc	*B. breve* JCM 1192^T genome (WT)
30	Downstream region of *rafBCD* (for pMSK197)	aaaggagtcaacaataggaaagcccagtgggct<br>atttaatgggaattccttgcggggacctgtaga	*B. breve* JCM 1192^T genome (WT)
31	Upstream region of BBKW_1838 (for pMSK197)	gtatatatgagtactcagcgtgaggcagctcc<br>gattactcctttgtgtgtcatgaattac	*B. kashiwanohense* JCM 15439^T genome (WT)
32	Downstream region of BBKW_1838 (for pMSK197)	cacaaaggagtaatccatccggttgcccgcca<br>atttaatgggaattcctgcatctgttcgccggtg	*B. kashiwanohense* JCM 15439^T genome (WT)
33	Upstream region of BBPC_1775–1777 (for pMSK197)	gtatatatgagtactttctgcagcgccgccatg<br>agtttttctctttcttcttaacccgtct	*B. pseudocatenulatum* JCM 1200^T genome (WT)
34	Downstream region of BBPC_1775–1777 (for pMSK197)	agaaagagaaaaactcatccggttgcccgcca<br>atttaatgggaattcctgcatctgttcgccggtg	*B. pseudocatenulatum* JCM 1200^T genome (WT)

Table S1. (Continued)

35	Upstream region of *pulP* (for pMSK197)	gtatatatgagtactctcaaaaccggccaggtg<br>tgttctccgttctcatctgtga	*B. adolescentis* JCM 1275^T genome (WT)
36	Downstream region of *pulP* (for pMSK197)	tgagaacggagaacagaccataatcgtctgactgc<br>atttaatgggaattccgagcaggtcctcccact	*B. adolescentis* JCM 1275^T genome (WT)
37	Upstream region of *aldh* (for pMSK197)	gtatatatgagtactaatccgtttccgtgaccacc<br>ggtgatgtcctttcgtaatactcgatag	*B. infantis* JCM 1222^T genome (WT)
38	Downstream region of *aldh* (for pMSK197)	cgaaaggacatcaccactcgtgcgtgaccactg<br>atttaatgggaattcacgctcatatccttatgcct	*B. infantis* JCM 1222^T genome (WT)
39	Upstream region of Blon_1092 (for pMSK197)	gtatatatgagtacttccgccgaactcgtgc<br>atccttatgcctccaaccggga	*B. infantis* JCM 1222^T genome (WT)
40	Downstream region of Blon_1092 (for pMSK197)	tggaggcataaggatcccatggcgcatacagac<br>atttaatgggaattctggcggaggcgatctgc	*B. infantis* JCM 1222^T genome (WT)
41	Genomic region comprising the up- and downstream 1.0-kb regions of *cpsD*	gactgtgggacgagatcg<br>ggcttcatcctggctgaac	*B. longum* JCM 31944 genome (WT, FCO, and SCO)
42	Genomic region comprising the up- and downstream 1.0-kb regions of BL105A_0502	gctactccgacttctacacc<br>cggcaatcagtgagatcagc	*B. longum* JCM 31944 genome (WT, FCO, and SCO)
43	Genomic region comprising the up- and downstream 1.0-kb regions of *rafBCD*	gccaccagcgatcccaatc<br>ccaccaggtccatcacgatc	*B. breve* JCM 1192^T genome (WT, FCO, and SCO)
44	Genomic region comprising the up- and downstream 1.0-kb regions of BBKW_1838	gagatcattgaacgcgaagatgg<br>gtgagggcagaccttgacg	*B. kashiwanohense* JCM 15439^T genome (WT, FCO, and SCO)
45	Genomic region comprising the up- and downstream 1.0-kb regions of BBPC_1775–1777	gaagatggcggttggtcg<br>gagcaggtattcgaggacga	*B. pseudocatenulatum* JCM 1200^T genome (WT, FCO, and SCO)
46	Genomic region comprising the up- and downstream 1.0-kb regions of *pulP*	gacgttccatgaacgacatctg<br>ctcggtgacggacaggtag	*B. adolescentis* JCM 1275^T genome (WT, FCO, and SCO)
47	Genomic region comprising the up- and downstream 1.0-kb regions of *aldh*	catcttgaagccacttacttcgtg<br>tgatgtggctcttgcccatc	*B. infantis* JCM 1222^T genome (WT, FCO, and SCO)
48	Genomic region comprising the upstream and downstream 1.0-kb regions of Blon_1092	tcgtgcagaccatcctgtg<br>gatgaccttgcggaagacg	*B. infantis* JCM 1222^T genome (WT, FCO, and SCO)
49	Vector linearization for In-Fusion cloning	gaattcccattaaataataaaac<br>agtactcatatatactttagattg	pMSK197 and pKO403

[a] The underlines indicate the 15 bp for In-Fusion cloning, while the double underline indicate the modified *clpP* terminator sequence [S2]. The uppercase letters indicate the three nucletides used for the substitution of the amino acid residue.

[b] FCO: first-crossover recombinant strain; SCO: second-crossover recombinant strain

Table S2. Mass spectrometric condition of aromatic lactic acids and internal standard, related to STAR Methods

Name	Polarity	Precursor ion (*m*/*z*)	Product ion (*m*/*z*)	Collision energy (V)
Phenyllactic acid (PLA)	Negative	164.95	119.1	18
4-Hydroxyphenyllactic acid (4-OH-PLA)	Negative	180.95	163.05	12
Indolelactic acid (ILA)	Negative	203.9	158.1	15
3-Methyl-2-oxindole (internal standard)	Positive	147.95	133.05	–25

References for Supplementary Information

S1. Sakaguchi, K., He, J., Tani, S., Kano, Y., and Suzuki, T. (2012). A targeted gene knockout method using a newly constructed temperature-sensitive plasmid mediated homologous recombination in *Bifidobacterium longum*. Appl Microbiol Biotechnol *95*, 499–509. 10.1007/s00253-012-4090-4.

S2. Ruiz, L., Motherway, M.O.C., Lanigan, N., and van Sinderen, D. (2013). Transposon Mutagenesis in *Bifidobacterium breve*: Construction and Characterization of a Tn5 Transposon Mutant Library for *Bifidobacterium breve* UCC2003. PLoS One *8*, e64699. 10.1371/journal.pone.0064699.
